# Recent advances on high-efficiency of microRNAs in different types of lung cancer: a comprehensive review

**DOI:** 10.1186/s12935-023-03133-z

**Published:** 2023-11-20

**Authors:** Mohammad Saleh Sadeghi, Mohadeseh lotfi, Narges Soltani, Elahe Farmani, Jaime Humberto Ortiz Fernandez, Sheida Akhlaghitehrani, Safaa Hallol Mohammed, Saman Yasamineh, Hesam Ghafouri Kalajahi, Omid Gholizadeh

**Affiliations:** 1 Department of Pharmaceutics, Faculty of Pharmacy, Tehran University of Medical Sciences, Tehran, Iran; 2 School of Medicine, Tehran University of Medical Science, Tehran, Iran; 3 School of Allied Medical Sciences, Tehran University of Medical Sciences, Tehran, Iran; 4 Tehran University of Medical Sciences, Tehran, Iran; 5 Universidad Peruana Los Andes, Huancayo, Peru; 6 Faculty of Medicine and Surgery, University of Rome Tor Vergata, Rome, Italy; 7 Collage of Pharmacy, National University of Science and Technology, Dhi Qar, Iraq; 8 Free Researcher, Viro & Biotech, Tehran, Iran; 9 Department of Biotechnology, Institute of Science, Uskudar University, Istanbul, Turkey

**Keywords:** NSCLC, SCLC, MicroRNA, Lung cancer, Treatment

## Abstract

Carcinoma of the lung is among the most common types of cancer globally. Concerning its histology, it is categorized as a non-small cell carcinoma (NSCLC) and a small cell cancer (SCLC) subtype. MicroRNAs (miRNAs) are a member of non-coding RNA whose nucleotides range from 19 to 25. They are known to be critical regulators of cancer via epigenetic control of oncogenes expression and by regulating tumor suppressor genes. miRNAs have an essential function in a tumorous microenvironment via modulating cancer cell growth, metastasis, angiogenesis, metabolism, and apoptosis. Moreover, a wide range of information produced via several investigations indicates their tumor-suppressing, oncogenic, diagnostic assessment, and predictive marker functions in different types of lung malignancy. miRNA mimics or anti-miRNAs can be transferred into a lung cancer cell, with possible curative implications. As a result, miRNAs hold promise as targets for lung cancer treatment and detection. In this study, we investigate the different functions of various miRNAs in different types of lung malignancy, which have been achieved in recent years that show the lung cancer-associated regulation of miRNAs expression, concerning their function in lung cancer beginning, development, and resistance to chemotherapy, also the probability to utilize miRNAs as predictive biomarkers for therapy reaction.

**Graphical abstract:**

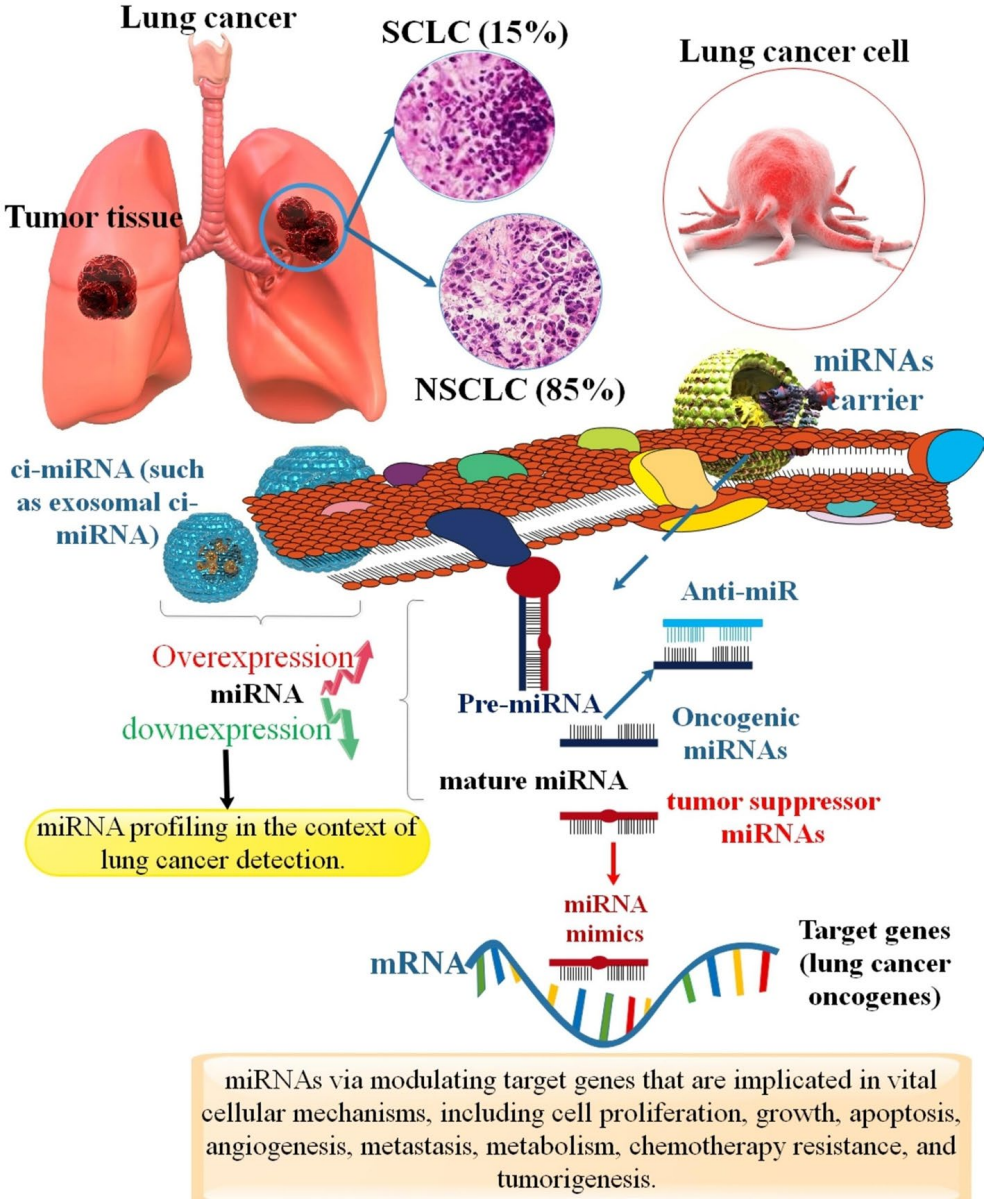

## Introduction

**Fig. 1:**
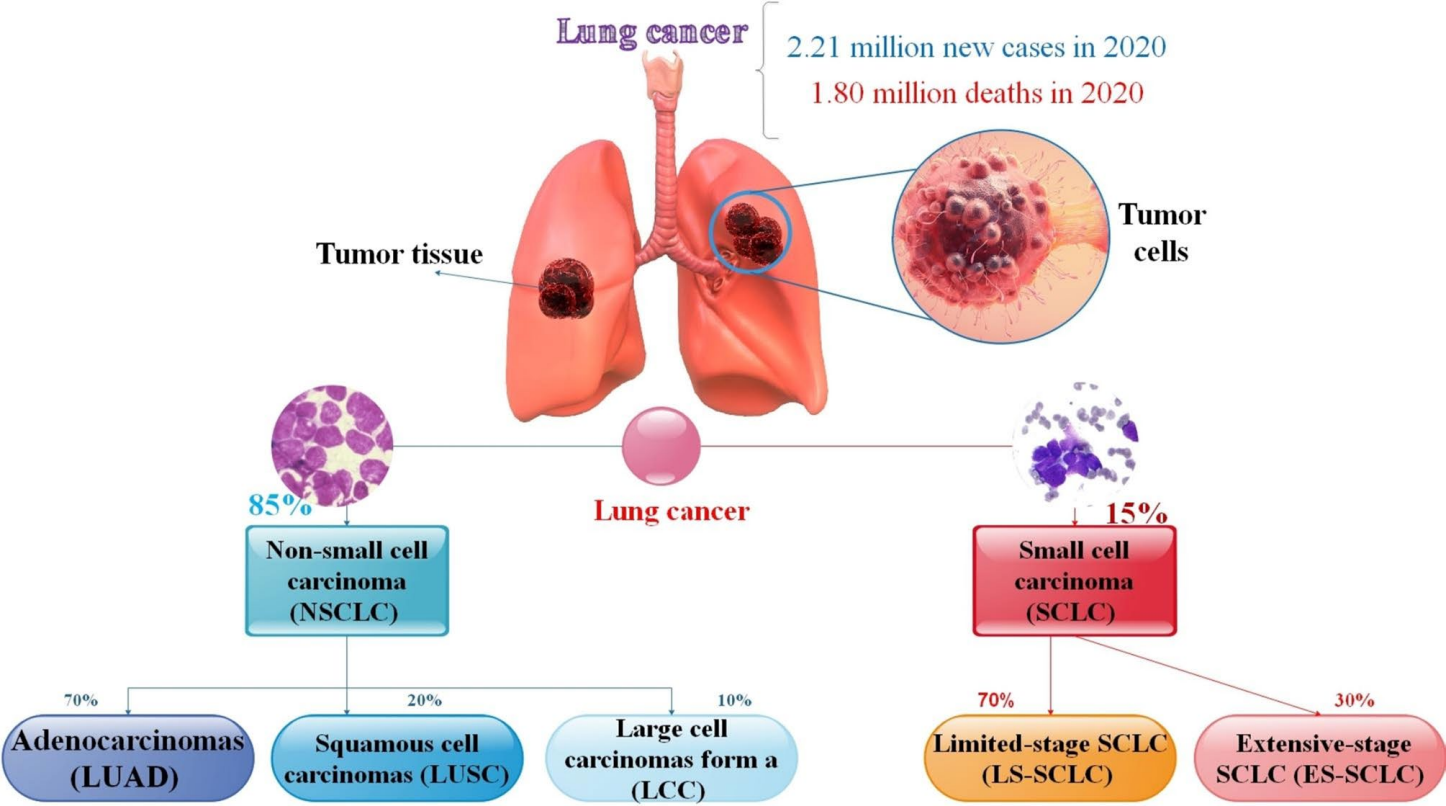
The histological classification and statistical analysis of lung cancer

According to a World Health Organization (WHO) report, cancer is a non-contagious disease responsible for 63% of mortalities in the world. Lung malignancy is the critical reason for cancer fatality globally and has the highest death rate among all types of cancer. Lung cancer results in rapid metastasis and is considered a major killer. It is assessed that about 236,740 novel patients of lung malignancy will be recognized in the USA in 2022, and approximately 130,180 cases of lung cancer will lose their lives [1–3]. Histologically, this malignancy is categorized into two groups: small-cell lung cancer (SCLC) and non-small-cell lung cancer (NSCLC) [4]. Approximately 14% of new diagnoses of cancer happen to be lung carcinoma, and about 70% of these newly created diagnoses turn out to be adenocarcinomas (LUAD) [5]. SCLC accounts for approximately 15% of all lung tumors, and about 85% are NSCLC. SCLC is categorized into limited-stage (LS-SCLC) and extensive-stage (ES-SCLC). The LS-SCLC cat includes about 30% of SCLC patients, and ES-SCLC comprises about 70% of all SCLC patients [6, 7]. LUAD forms the majority of NSCLCs (LUAD ≈ 70%), and squamous cell carcinomas second them (LUSC ≈ 20%). Also, large cell carcinomas form a small minority (LCC ≈ 10%) [8, 9]. The majority of NSCLCs are genetically intricate tumors harboring numerous possible activating events. Genomic alterations (mutation, rearrangement, amplification) in NSCLC include (Epidermal growth factor receptor) EGFR, ALK, ROS1, RET, BRAF, FGFR-1, MET, DDR2, PIK3C, AKT, and PTEN (Table 1). Together with the mutation of EGFR and rearrangement of ALK, they account for approximately 20% of NSCLCs. EGFR is the best-appointed driver mutation in NSCLC [10–13]. In a study, researchers showed that the number of mortalities associated with lung cancer (NSCLC) in China may increase to about 40% in 2030. Therefore, there is an essential and urgent requirement to find novel and safe detection and treatment methods for lung cancer [14] (Fig. 1).

**Table 1:** Genomic alterations in NSCLC

Name of Target	Location	Type of genomic alterations	Function	Explanation	Target miRNAs in lung caner	Ref.
EGFR (Epidermal growth factor receptor)	7p11.2	Mutation	EGFR is a cell signaling, transmembrane protein intimately implicated in cell proliferation.	The occurrence is maximum in females and non-smokers, with LUAD histology, 90% of patients in LUAD.	miR-128b, miR-545-3p, and miR-494-3p	[15–17]
ALK (Anaplastic lymphoma kinase)	2p23.2-p23.1	Rearrangement	Gene productions are recognized to increase cell growth/proliferation and suppress apoptosis at baseline.	It was detected in 5–7% of NSCLC, mostly in never-smokers and approximately exclusively in LUAD.	miR-100-5p, and miR-200 C	[18, 19]
ROS1	6q22.1	Rearrangement	This gene increases proliferation and suppresses apoptosis.	It was detected in tumors, which are 1–2% of all NSCLCs. It preferentially impacts young persons, never-smokers with LUAD.	miR-760	[20]
KRAS	12p12.1	Mutations	The proto-oncogene generated was initially found to be a GTPase p21.	About 25% of smokers related to LUAD.	miR-181a-5p, miR-199b, miR-29b, let-7, and miR-21	[21–24]
RET	10q11.21	Rearrangement	A tyrosine kinase responds to growth factors and increases cell proliferation.	In 1–2% of lung LUAD.	miR-182	[25]
BRAF	7q34	Mutation	BRAF is a member of the Raf kinases, which modulate the MAP kinase pathway.	BRAF mutations are identified in 1–4% of LUAD.	miR-21, and miR-130a-3p	[26, 27]
FGFR-1 (Fibroblast growth factor receptor-1)	8p11.23	Amplification	Have function in cell proliferation	Found in about 15–20% of LUSCs, and amplification is related to smoking and with worse overall survival.	Hsa-miR-16-1, miR‑497, and miR-214-3p	[28–30]
MET	7q31.2	Amplification	Encodes a protein production named the HGF receptor (HGFR) and its role in promoting tumor growth, development, and invasion in many cancers.	MET amplification is identified in 2–4% of untreated NSCLC.	miR-34a/c-5p, miR-449a/b, miR-206. and miR-182	[31–33]
DDR2 (Discoidin death receptor 2)	1q23.3	Mutation	Tyrosine kinase action and mutation of this gene in NSCLC are implicated in increased cell migration, development, and survival.	In 2.2–3.8% of LUSC smokers, in LUAD and LUSC patients, had one single type of KRAS mutation.	miR-30c, and miR-15a-5p	[34, 35]
PIK3CA (phosphatidylinositol-4,5-bisphosphate 3-kinase catalytic subunit alpha)	3q26.32	Mutation	Lipid kinases are involved in the modulation of cell growth, development, and survival.	Occurs in less than 5% of NSCLCs.	miR-124-3p, miR-142-5p, and miR-183-5	[36–38]
AKT1 (AKT serine/threonine kinase 1)	14q32.33	Mutation	Proliferation, survival, migration, and invasion.	Great rates of intra-tumoral AKT2 in NSCLC and AKT1 in LUAD.	miR-99a, miR-9500, and miR-548 L	[39–41]
PTEN (phosphatase and tensin homolog)	10q23.31	Mutations	The tumor suppressor gene is an essential function in cell cycle development, apoptosis, growth, proliferation, and migration.	This gene protein expression is relatively usual in LUSC.	miR-10a, miR-21, and miR-20a	[42–44]

MicroRNAs (miRNAs) are short ss-RNA endogenous critical preserved non-coding RNAs, which have important regulatory roles in cellular and physiological happenings, including proliferation, cell development, neoplastic transformation, cell renewal, and repair [45, 46]. Discoveries have demonstrated a close link between miRNA expression and the progress of several illnesses, showing the significance of miRNA as clinical molecular markers and objectives for medication identification [47–50]. Augmenting proof offers that miRNA-based treatments, either restoring or suppressing miRNA expression and acting, hold excellent promise [51, 52]. More than 1000 miRNAs are related to human diseases, such as cancers. Relative constancy, small dimensions, and significant modulation of gene expression are the unique properties of miRNAs that distinguish miRNAs from mRNAs and recognize them as novel and robust curative biomarkers [53, 54]. Calin et al. were the first ones to discover an acceptable interdependency between miRNA dysregulation and tumors in 2002. After two years, Junichi Takamizawa et al. found overexpression of miRNAs (including let-7) in ATCC® CCL-185™ lung LUAD cell culture suppressed cancer cell growth in vitro. Consequently, they found a relationship between miRNA expression and lung malignancy. The miRNAs, in the context of lung cancer, have a vital function in the diagnosis of the stage and kind of cancer [55–57]. MiRNAs regulate protein expression in cellular activity, including cell development, cell-division cycle, posttranscriptional, translational phase, proliferation, and apoptosis, which are essential in maintaining the status quo of the cell [58]. The miRNA expression levels may be dissimilar among localized lung cancers, metastasized lung cancers, and solid tumors, which result in a differing rate of metabolic deregulation [59, 60]. Recently, several investigations showed that serum/plasma miRNA expression levels could efficiently diagnose cancer patients and healthy persons and might be associated with the progress of lung cancer or might have clinical capability importance in the initial detection, prediction, and curative of lung cancer [61, 62].

In this study, we investigate the different functions of various miRNAs in different types of lung tumors, which have been achieved in recent years that show the lung cancer-associated regulation of miRNAs expression about their role in lung cancer beginning, development, and resistance to chemotherapy, also the probability to utilize miRNAs as predictive biomarkers for therapy reaction.

## miRNAs biogenesis and function

**Fig. 2:**
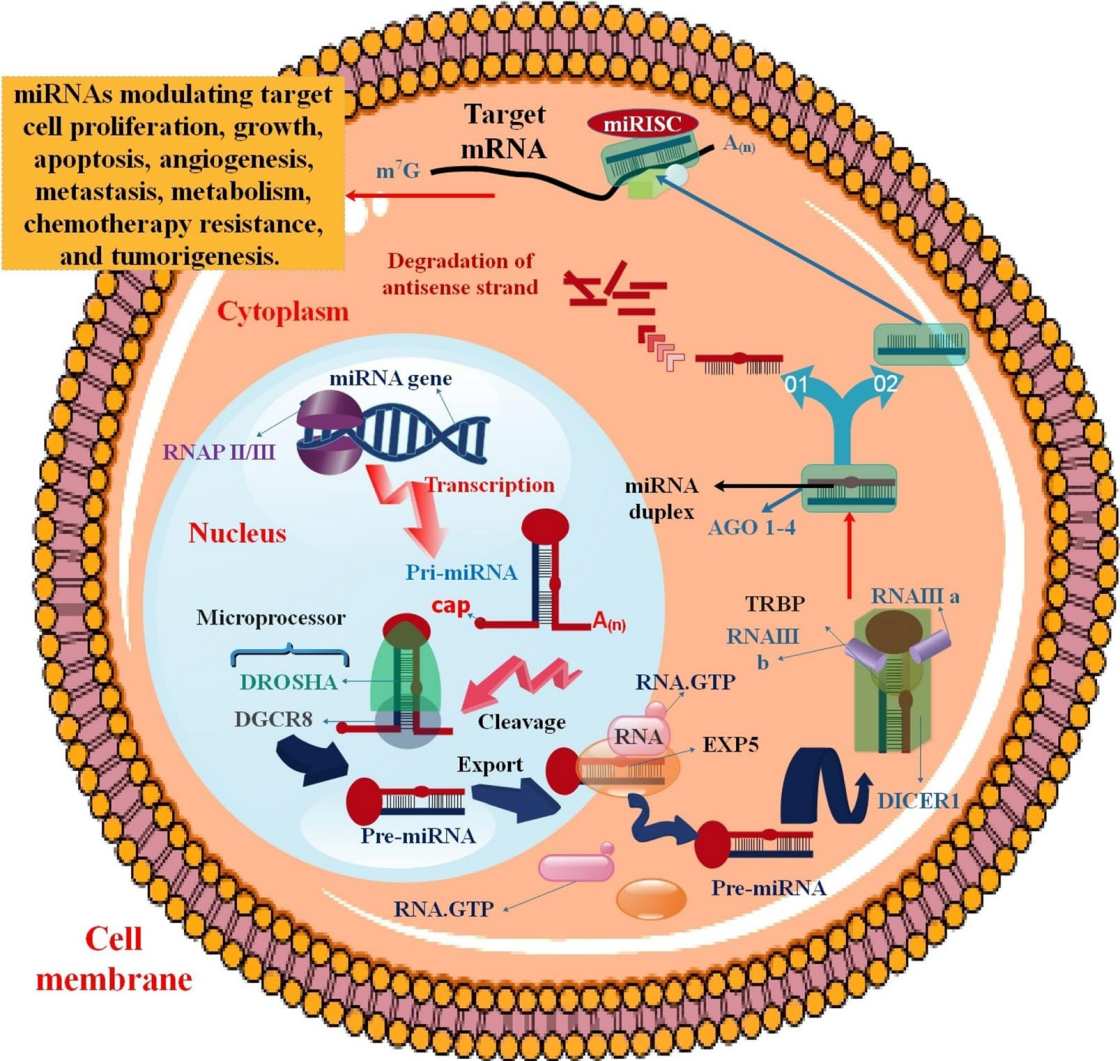
The process by which microRNAs are formed. The pre-miRNA loop is cleaved in the cytoplasm by a triple combination of small miRNA duplexes, Dicer, and TRBP

miRNAs are small single-stranded (ss) ncRNAs, about 19–25 nucleotide length. miRNA genes are transcribed via RNAP II. About 40% of miRNA genes happen to be positioned in the intron areas [63, 64]. Transcription within the nucleus, which gives rise to pre-miRNAs as well as pro-miRNAs, then hairpin formed in the nuclear processing, which results in a 70–100 nucleotide. This processing is completed via the microprocessor complex. This complex is minimally constituted of the Drosha and the dimeric RNA-binding protein DiGeorge Critical Region 8 (DGCR8) (as well as recognized as Pasha in non-human animals) and cleaves pri-miRNA to pre-miRNA in the cell nucleus [65–67]. Pre-miRNA hairpins are transported from the nucleus to the cytoplasm via a RanGTP/exportin 5-dependent method [68, 69]. Following this process, the pre-miRNA hairpin is cut via Dicer and converts the pre-miRNA into a mature double-stranded (ds) ~ 22 nt miRNA/miRNA duplex. The mature ss-miRNAs connect to the RNA-induced silencing complex (RISC). This complex contains proteins of the Ago1 to Ago4 in humans and transactivation response element RNA-binding protein (TRBP) [70]. The 5′ terminals of mature miRNAs have seed sequences (approximately eight nucleotides) that identify specific mRNA [71]. A particular miRNA can suppress more than a hundred mRNAs. The inhibition process of miRNAs includes (1) elongation suppression (mRNA cleavage or repressed mRNAs), (2) inhibition of translation (Cap and 60 S joining inhibition), (3) ribosome drop-off, i.e., premature termination, and (4) Co-translational protein degradation [72] (Fig. 2). miRNAs play diverse roles in various diseases. The miRNA, as a novel strategy in the diagnosis and therapy of cancers, has produced high attentiveness for clinicians. The upregulation and downregulation of miRNAs contribute to the pathogenesis of different human malignancies via controlling target genes implicated in vital cellular mechanisms, including cell proliferation, growth, apoptosis, angiogenesis, metastasis, metabolism, chemotherapy resistance, and tumorigenesis [73, 74]. The conjunction of miRNAs into multivesicular bodies is not an accidental incident. The sorting process can precisely guide intracellular miRNAs into exosomes via five mechanisms. These mechanisms include the miRISC-associated pathway, the neural sphingomyelinase 2 (nSMase2)-associated pathway, the pathway related to the 3′-end adenylation and uridylation of miRNAs, the pathway linked to miRNA motifs and sumoylated heterogeneous nuclear ribonucleoproteins (hnRNPs), and the ceramide-associated pathway [75]. The diagnostic, prognostic, and therapeutic potential of using these miRNA signatures for lung cancer categorization has been shown. According to the available data, miRNAs have promise as new entry points for lung cancer diagnosis, prognosis, and treatment. Furthermore, it has been evident during the last ten years that human malignancies have dysregulated miRNA expression. The underlying processes, which are discussed in the section that follows, include chromosomal anomalies, alterations in transcriptional regulation, epigenetic modifications, and malfunctions in the machinery that produces miRNA.

## miRNA deregulation, mechanisms, and pathways

Any alterations in the sequences of miRNAs, whether they are mature, precursor, or primary transcripts, may contribute to the pathogenesis of malignancies [76] (Table 2). miRNAs can activate numerous signaling pathways to produce various efficacies. According to these effects, they can be categorized into two principal kinds: tumor-suppressive miRNAs and oncogenic miRNAs (oncomirs) [77]. Many miRNAs promote the formation of a tumor and cancer development via enhancing tumor progression, angiogenesis, invasion, and tolerance of host immune response, while others have tumor suppressive efficacy [78]. In addition, the adverse events of mutations can be suppressed via a functional miRNA system, and the change of the miRNA machinery lacks significant outcomes in the absence of genotoxic degradation [79, 80]. Besides, several investigations have displayed that miRNAs are effectively involved in each physiological procedure, and thus, miRNA regulatory disorders have been associated with numerous human pathologies. Therefore, miRNA mimics and miRNAs antagonistic that improve miRNA expression or downregulate inappropriately expressed miRNAsare incredibly considered-desired curative methods for efficient manipulation of miRNA rates [81, 82]. For example, miR-146a is an essential modulator of innate immune reaction sequences. In addition, it has been shown that miR-146a may have a significant function in multiple hallmarks of lung cancer, including escaping growth obstacles, cell reproduction, resisting apoptosis, increasing angiogenesis, inflammation, cancer immune tolerance, Warburg effect, metastasis, and genome fragility [83]. In another study, researchers showed that miR-133b suppresses cell growth, migration, and invasion in NSCLC. In addition, this miRNA downstream target NCAPH can compete with Akt1 to form a complex with β-catenin, which prevents β-catenin phosphorylation and ubiquitin interceded protein destruction, resulting in triggered Wnt signaling and a more favorable niche for cancer stem cells in NSCLC [84]. In addition, different miRNAs, such as miR-619-5p, were related to angiogenesis and metastasis, and the presentation of miR-1260b or exosomal miR-1260b stimulated migration and invasion or resistance to cisplatin in NSCLC cells. Furthermore, several investigations showed that miR-1260b could elicit angiogenesis by VEGF discharge in cancer cells [85]. In a study, investigators showed that decreased miR-192-5p and enhanced TRIM44 rates related to the proliferation and metastasis of lung cancer. Furthermore, miR-192-5p upregulation in NSCLC cell lines suppresses NSCLC metastasis, presumably via downregulating TRIM44 [86]. Deregulation of miRNA expression leads to their functional alterations. Genetic loss, epigenetic modifications, wide-spreading transcriptional suppression, or faulty biogenesis are the significant reasons for aberration in mature miRNA rate in the case of lung cancer. miRNAs have a specific potential to regulate the complex network of gene modulation in several lung cancer-associated pathways, such as cell proliferation and cell cycle control, apoptosis, p53, invasion, and metastasis [87]. The state of active signaling pathways may be influenced by microRNAs because of their ability to modulate gene expression. Cancer prevention, diagnosis, and therapy might benefit from a more profound knowledge of miRNA dysregulation and its interplay with epigenetic pathways. Epigenetic alterations in DNA methylation and miRNAs have been linked to a subset of improperly expressed genes in lung cancer. As we have shown, this is supported by the high frequency of aberrantly methylated miRNA genes linked to the prognosis of lung cancer and may be used as biomarkers for precise lung cancer diagnosis and therapy. Furthermore, miRNAs may have a direct or indirect function in lung cancer cells’ metabolism of lipids, amino acids, and glucose. Next, we show how miRNAs regulate transcription factors, signaling pathways, and enzymes in lung cancer cell metabolism and mitochondrial calcium import/export processes.

**Table 2:** Pathways of miRNA dysregulation in lung cancer [88]

Mechanisms of miRNA	Explain	Example
Genomic abnormalities	Approximately 25% of miRNA genes are placed in chromosomal breakpoints, fragile sites, and areas lacking heterozygosity (LOH) or amplification. miRNAs are susceptible to genomic changes.	In lung cancer, a minimum LOH area of 17p13was nearly (1.9 Mb) equaled with the hsa-miR-22 cluster, and a place of homozygous deletion on 21q11 was near (2.8 Mb) the hsa-let-7c cluster [89].
Epigenetic modifications	DNA methylation and histone modifications could inhibit anti-oncogenes and subscribe to cancer beginning and development.	MiR-9/3, miR-193a, and miR-34b/c were the objectives of epigenetic inhibiting via DNA methylation in lung cancer [90, 91].
Polymorphisms of miRNAs	Single nucleotide polymorphism (SNP) between the miRNA nucleotides	SNP (LCS6) in the 3′-UTR of the KRAS gene, which modified the desire to connect of let-7, was identified to be related to lung tumor danger in low-intensity cigarette smoking [92].
Transcriptional regulation	Pri-miRNA transcription is modulated via transcription agents or genes that are dysregulated in the tumor.	Myc and E2F transcription factor 1 was identified to impact the expression levels of the oncomiR miR-17-92 cluster [93].
Abnormal maturation pathways	Abnormality or impairment in the function of Drosha and Dicer in the procedure of immature pri-miRNAs into a pre-miRNA.	Knockdown of KH-type splicing regulatory protein (KSRP), which improves these RNase III enzymes, could suppress the expression of let-7a and miR-206 and thus impact cell proliferation and differentiation [94].

## miRNA in lung Tumor metabolism

**Fig. 3:**
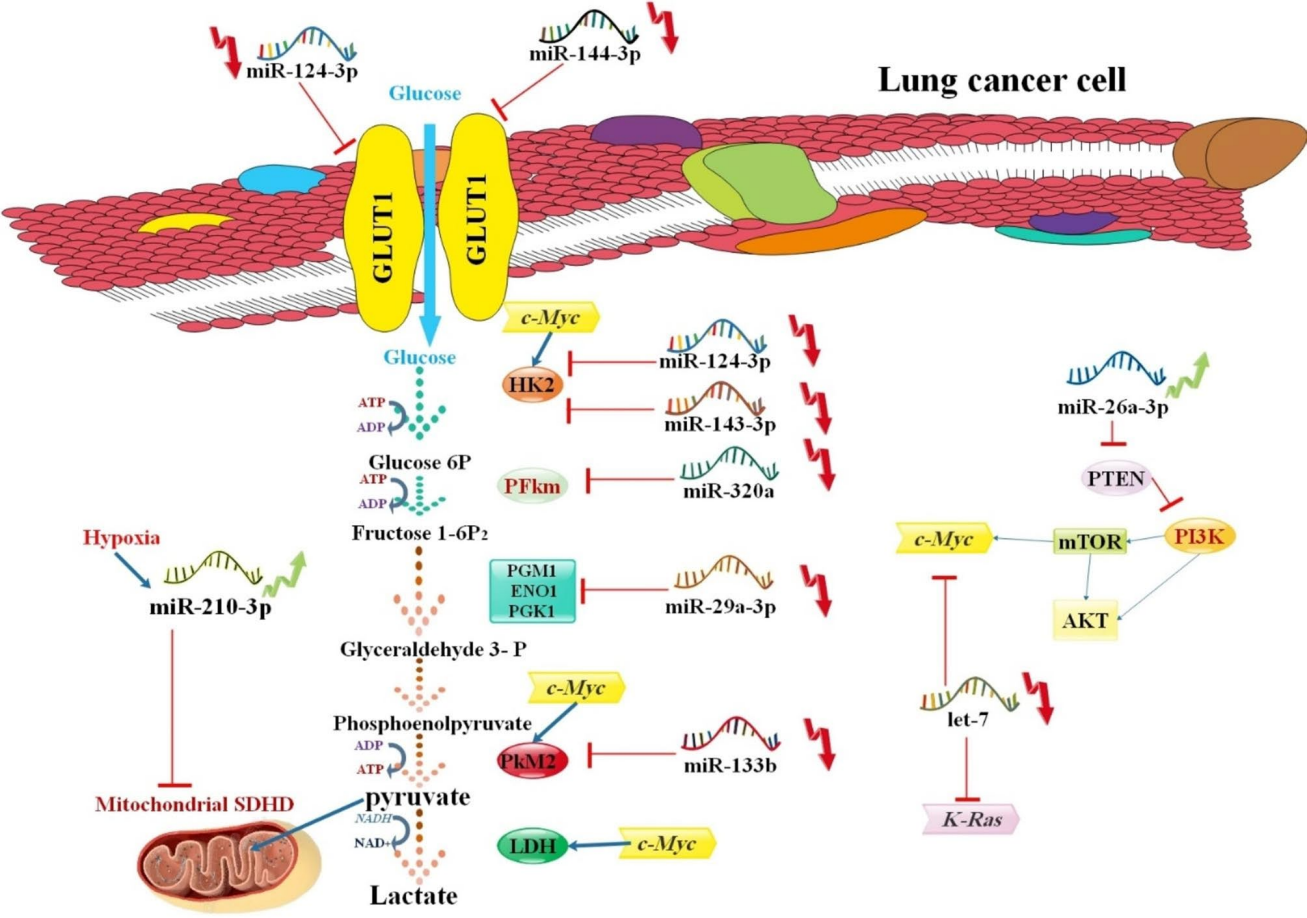
miRNAs and Warburg effect of lung tumor. This illustration explains the down/upregulated expression of miRNAs and its impact on lung tumor cell metabolism

The metabolism of tumor cells was explained approximately a century ago as an unusual conformity of cancer cells first to metabolize glucose converted to lactate in a hypoxic situation, recognized as the “Warburg effect” [95]. The transformed glucose metabolism frequently observed in tumor cells has been used for malignancy detection and therapy. Such metabolic change is essential for intermediates and different pathways for energy production and macromolecular synthesis to support the proliferative behavior of cancer cells [96]. miRNAs have significant functions in modulating cancer metabolic processes, including directly affecting the expression of essential enzymes of glycolysis, indirectly controlling oncogenes/tumor suppressors, several oncogenic signaling pathways, and regulating transcription factors [97]. For example, miR-143-5p per se targets the expression of enzymes of glycolysis in human lung cancer. This miRNA downregulated expression of glycolytic enzyme Hexokinase 2 (HK2), which gene is frequently overexpressed in malignant tumors by mammalian target of rapamycin activation, decreases glucose metabolism, prevents cancer cell development, and inhibits tumor development over targeting HK2 [98]. MiR-144-5p function in the metabolism of the lung cancer cells is contrary to miR-143-5p functions, and miR-144-5p regulates the expression of Glucose transporter (GLUT1) by connecting its 3′UTR, consequently decreasing the expression of miR-144-5p and increases the glucose metabolism of lung tumor cells [99]. MiR-124-5p expression in NSCLC, via targeting Protein Kinase B1/2, stops the expression of GLUT1 and HK2 [100]. MiR-320a, by downregulation of muscle-type phosphofructokinase (PFK) in lung cancer, can regulate glucose metabolism. Subsequently, this miRNA modulates oxidative stress-stimulated PFKm expression and decreases the miR-320a, causing more stimulation of glycolytic enzyme in reaction to H2O2 treatment [101]. In another study, hypoxia-regulated miRNA (miR-210-3p) was displayed to control the mitochondrial metabolism of LUAD A549 cells. This miRNA regulates the expression of subunit D of succinate-coenzyme Q reductase (SQR), an enzyme compound usually recognized for its function in the Krebs cycle. It controls the mitochondrial metabolism and Hypoxia-inducible factor 1 (HIF 1) function to elevate glycolysis [102] (Fig. 3). In a different study, NSCLC serum samples showed low expression of miR-199a-5p. In NSCLC cell lines, miR-199a-5p inhibited cell migration, proliferation, and cell cycle arrest. The findings indicated that the direct target of miR-199a-5p was SLC2A1 (glucose transporter 1, or GLUT1). SLC2A1 downregulation may enhance apoptosis and prevent migration, proliferation, and cell cycle progression. Based on the findings, it seems that miR-199a-5p may target SLC2A1 and, hence, limit glucose metabolism in NSCLC. By focusing on SLC2A1, this research demonstrates how miR-199a-5p / SLC2A1 might be crucial in developing NSCLC [103]. With progressions in our understanding of miRNAs, the importance of miRNAs in cancer glucose metabolism has attracted much attention owing to their enormous therapeutic capability. Emerging investigations have demonstrated that miRNA can affect cancer metabolism by directly controlling the expression of main glycolytic enzymes and indirectly by maintaining the oncogenic or tumor suppressor signaling pathways and transcription factors that regulate glucose metabolism [87].

Recent data has shown the connection between lung cancer metabolic reprogramming and the differential expression of miRNAs in sustaining carcinogenesis. By focusing on essential enzymes, transporter proteins, or regulatory elements in addition to metabolic signaling pathways, this section sheds light on the roles played by various miRNAs in lung cancer metabolic reprogramming. Intrinsic apoptotic signals are the only regulators of mitochondria-mediated apoptosis. Apoptosis is triggered in cells that experience oncogenic stress, DNA damage, and unchecked growth. Therefore, the best therapy for lung cancer is the induction of apoptosis, which stops cell proliferation. The kinds of microRNAs that are involved in the treatment of lung cancer (both SCLC and NSCLC) are discussed in the section that follows.

## Different miRNAs associated with Lung cancer

The first research on Caenorhabditis elegans and then in Drosophila, with the detection of lin-4 and let-7, reported a link between cancer and miRNA. Knockout of lin-4 or let-7 in C. elegans, whereas Bantam upregulation in Drosophila led to cellular development and the inhibition of programmed cell death [104]. Most miRNAs are utilized in lung malignancy (NSCLC and SCLC) as tumor markers and therapeutic measures. Increases or decreases in miRNA expression level can distinguish the kind of lung tumor. miRNAs can increase or decrease the expression of genes associated with lung tumors. The expression of some miRNAs associated with lung tumors is enhanced. In addition, a fundamental therapy for lung cancer (LC) should target the crucial regulatory and coding sequences implicated in the pathogenesis of LC. miRNAs are greatly proficient in controlling the expression of non-coding sequences and other numerous genes involved in controlling signal cascades regulating tumorigenesis. Therefore, miRNAs can be used as potential targets for lung cancer therapy [87]. For example, by controlling the Ras family, let-7 regulates cellular proliferation in vivo models. However, treatment with this miRNA demonstrated different mechanisms with downstream triggering that result in resistance of cancer cells to the tumor-suppressive roles of this transcript. In NSCLC, let-7 low expression was remarkably related to patient outcome. However, some investigations were not successful in establishing a direct association with low expression of let-7 and prognostic of NSCLC patients [105]. MiR-21 shows an intensively investigated miRNA in many types of cancer, such as NSCLC. Its act as an oncogene is underlined in several investigations reporting the upregulated expression of this sequence in patients diagnosed with this cancer; furthermore, multiple investigations related to this enhanced expression of miR-21 have a worse outcome within NSCLC patients [106]. In a study, researchers prepared a method to capture miRNAs based on circular RNAs (circRNA). To show its effectiveness, researchers chose miR-21, which is upregulated and involved in LC. Circ-21 stimulated a considerable and time-related reduction in the growth of A549 and LL2 cells but not in L132 cells. Moreover, A549 and LL2 cells transfected with Circ-21 demonstrated fewer colonies and migration than L132. Similar findings were observed in A549 and LL2 Circ-21 multicellular tumor spheroids, demonstrating a considerable reduction in volume growth but not in L132 Circ-21 MTS. Based on this, the miR-21 circular sponge may inhibit the processes of tumorigenesis and development [107]. In addition, the efficacy of cooperative control of miR-21 and let-7 on lung cancer is higher than that of a single miRNA. MiR-21 and let-7 are crucial differentially expressed genes in lung cancer controlled through the K-ras pathway. As a result, for multigene LC, the cooperative control of two miRNAs will offer a novel target and direction for lung cancer treatment in the future [23].

The miR-17-92 gene cluster is an oncogene cluster that is characterized by its high conservation and encodes six miRNAs: miR-17, miR-18a, miR-19a, miR-19b-1, miR-20a, and miR-92a [108]. The miR-17-92 cluster has garnered significant attention in miRNA research due to its extensive investigation. Initially identified as an oncogene, further studies have shown its potential as a tumor suppressor in some human malignancies [109]. It was recently demonstrated that miR-18a-5p, a member of the miR-17-92 cluster, functions as an oncogene in lung cancer by directly repressing the expression of interferon regulatory factor 2. Furthermore, miR-17-5p expression was initially shown to be considerably higher in NSCLC patients compared to healthy controls, suggesting that miR-17-5p has diagnostic potential for NSCLC [110, 111].

Patients with lung adenocarcinoma, squamous cell carcinoma, and large-cell neuroendocrine carcinoma had higher levels of miR-31 expression than those with small-cell carcinoma or carcinoids but not normal lung tissue. Mice-bearing xenografts of human adenocarcinoma or squamous cell carcinoma cell lines showed increased tumor development when treated with miR-31 but not with large- or small-cell carcinoma cell lines. Though miR-31 did not affect original tumor development, it facilitated spontaneous metastasis in both large- and small-cell carcinomas. Each histologic subtype had a unique phenotype due to the various cellular signaling cascades that miR-31 regulated [112]. Additionally, the protein level of HuR was elevated, whereas miR-31 was lowered in lung carcinoma cells and lung cancer (LC) tissues. miR-31 aimed at and successfully hit HuR. HuR overexpression and miR-31 inhibition led to an increase in cyclins A2, B1, and D1 and vascular endothelial growth factor (VEGF). In addition, miR-31 overexpression caused lung cancer cells to commit suicide and prevented them from migrating [113].

The miR-200 family has exhibited various functions in several cellular processes, including cell proliferation, cancer progression, control of epithelial-mesenchymal transition (EMT), autophagy, and resistance to anti-cancer drugs. Numerous studies have shown the decreased expression of the miR-200 family in cancerous tissues and cancer cells that exhibit resistance to anti-cancer drugs [114–116]. The miR-200 family is a significant regulator of miR in the context of EMT and has been associated with poor overall survival (OS) in patients with LC. The tumor microenvironment (TME) is a multifaceted contributor to the advancement and dissemination of tumors. The research highlights the significance of the miR-200 family in regulating immune-related genes (IRGs) and its impact on LC, resulting in increased metastasis and worse OS among patients with LUAD. The effect of these immune-related genes (IRGs) may also have implications for the composition of tumor-infiltrating cells in LUAD since there seems to be a correlation between these immune effector cells and their potential involvement in tumor growth, invasion, and metastasis. Research on the C-X3-C motif chemokine ligand 1-C-X3-C motif chemokine receptor 1 (CX3CL1-CX3CR1) axis, which may be linked to the advancement of LC, shed light on the function of miR-200a–3p. For effective management of LUAD-related metastasis and poor survival, targeting the miR-200a-CX3CR1-Spi-B transcription factor (SPiB) and miR-141-C-X-C motif chemokine receptor 1- T-Box transcription factor 21 (miR-141-CXCR1-TBX21) axes may offer a potential prognostic as well as a therapeutic target [117].

From nematodes to humans, the let-7 family of miRNAs is among the most prominent and most conserved miRNA families. This family is made up of many paralog genes that are situated on separate chromosomes [118]. Target genes of let-7 include the cell cycle and proliferation-related genes c-Myc, high-mobility group A (HMGA), also known as signal transducer and activator of transcription 3 (STAT3), and Janus-activated kinase 2 (JAK2). Let-7 family transfection significantly lowered the proliferation rate in NSCLC cells, functioning as a tumor suppressor gene. In in vivo models, let-7 regulates the Ras family, which helps to control cellular proliferation. However, treatment with this miRNA has shown that other mechanisms with downstream activation cause activation that cause cancer cells to become resistant to the tumor-suppressive properties of this transcript. Studies have revealed a substantial correlation between let-7 under expression and patient outcome in NSCLC; however, no clear correlation has been found between low let-7 expression and the prognosis of NSCLC patients [105].

Loss of miR-101 expression is related to poor survival in patients with LC, and it is upregulated early in carcinogenesis in low-grade lung cancers. Tumor stroma is mainly composed of cancer-associated fibroblasts (CAFs), which promote tumor growth. Increasing apoptosis and inhibiting tumor cell proliferation, migration, and invasion resulted from of miR-101 overexpression in CAFs. More analysis revealed that miR-101 controlled the biological activity of cells by regulating the expression of C-X-C motif chemokine 12 (CXCL12). These results suggest a novel mechanism through which miR-101 down-regulation in CAFs may suppress lung cancer proliferation and metastasis by targeting CXCL12 [119]. Furthermore, by focusing on the Metastasis-Associated Lung Adenocarcinoma Transcript 1 (MALAT-1), MiR-101-3p blocked the PI3K/AKT signal pathway, hence reducing NSCLC development and metastasis [120].

Super-regulatory functions for miR-106a in essential activities are well-documented. Therefore, there is a lot of interest in studying its expression in illnesses as a means of molecular diagnostics and therapeutic development. Numerous research studies have looked into the genes targeted by miR-106 and found that this miRNA affects the expression of essential proteins involved in the cell cycle and apoptosis, pointing to miR-106a as a promising diagnostic and prognostic biomarker. The link between miR-106a expression and cancer medication resistance further illustrates the multifaceted nature of this molecule’s roles in many tissues [121]. In addition, new research indicates that miR-106a has an oncogenic function in the development of pancreatic tumors by facilitating the proliferation, EMT, and invasion of cancer cells by focusing on tissue inhibitors of metalloproteinase 2 (TIMP-2) [122]. Further, by reducing PTEN expression, miR-106a stifled NSCLC cell proliferation and invasion. These findings provide unique information that might be used to develop effective therapies for managing NSCLC [123].

MiR-15a is implicated in NSCLC metastasis, proliferation, invasion, and metastasis via targeting-regulating mothers against decapentaplegic homolog3 (Smad3) expression. This provides the theoretical groundwork for assessing the prognosis of NSCLC patients and researching the causes and mechanisms of NSCLC development [124]. MiR-195 belongs to the miR-15/16 family, which includes other miRNAs with the same seed sequence (miR-15a, miR-15b, miR-16-1, and miR-16-2). Non-small cell lung cancer (NSCLC) is only one cancer where the expression of miR-195 is related to improved prognosis. Tumor cell proliferation, migration, and invasion slowed when miR-195 was overexpressed. Researchers found that miR-195 directly targets Checkpoint kinase 1 (CHEK1), reducing CHEK1 expression in lung cancers. High levels of CHEK1 expression in lung cancers were linked to a shorter survival time. Based on these findings, miR-195 may inhibit NSCLC and serve as a prognostic indicator for lung cancer [125].

Because of its synergistic action with the well-known tumor suppressor p53, microRNA-34 (miR-34) is thought to be tumor-suppressive and has been documented to be dysregulated in several human malignancies. The importance of miR-34 is becoming increasingly apparent with the use of MRX34 in phase I clinical trials (NCT01829971), the first tumor-targeted microRNA medication based on miR-34a mimics. By interacting with EMT-related transcription factors, p53, and other key signaling pathways, miR-34 is critical in inhibiting tumor growth [126]. Results suggested that miR-34a and miR-34c might be used as prognostic indicators in NSCLC. In addition, miR-34b/c expression was lower in metastatic LUAD compared to non-metastatic LUAD, suggesting that miR-34b/c may inhibit metastasis capacity in LUAD cells, although miR-34a expression was not significantly different [127]. Additionally, methylation led to the down-regulation of miR-34a and miR-34b/c in SCLC. Methylation occurred more often with miR-34b/c than with miR-34a. These findings suggest that lung tissues are the primary sites where miR-34b/c exerts its effects [128, 129].

Increased miR-184 expression in the serum exosomes of people with NSCLC is associated with a worse prognosis, but it also has diagnostic utility in distinguishing between benign and malignant lung diseases [130]. Complement C1q Tumor Necrosis factor-related Protein 6 (C1QTNF6) overexpression in LUAD promoted malignant cellular activities. MiR-184 suppressed C1QTNF6 expression and was poorly expressed in LUAD. By focusing on C1QTNF6, miR-184 inhibited LUAD cell processes [131]. For instance, Lin et al. discovered that miR-184 functions as a tumor suppressor by inhibiting the proliferation and invasion of NSCLC cells by targeting cell division cycle 25 homolog A (CDC25A) and c-Myc [132].

The dysregulation of miR-574-5p has been seen in several pathogenic mechanisms associated with carcinogenesis, including cancer cell proliferation, migration, invasion, metastasis, apoptosis, EMT, and angiogenesis [133]. There is a differential expression of serum miRNAs seen between individuals diagnosed with early-stage NSCLC and a control group. Further validation is necessary to confirm the efficacy of miR-1254 and miR-574-5p serum-based biomarkers as minimally invasive screening and triage tools for future diagnostic assessment [134]. The significance of small extracellular vesicles (sEV) in facilitating intercellular communication is of considerable importance. The inflammatory lipid mediator prostaglandin E2 (PGE2) induces explicitly the expression of miR-574-5p in the sEV of A549 and 2106T cells. Researchers have made a significant finding indicating that miR-574-5p, a molecule generated by sEV, can stimulate Toll-like receptors (TLR) 7/8. This activation subsequently leads to a reduction in the levels of PGE2. Conversely, the intracellular miR-574-5p stimulates the formation of PGE2. Thus, PGE2 levels are regulated by a feedback loop involving miR-574-5p both inside cells and in sEVs. This was only seen in adeno- but not squamous cell carcinoma, suggesting that different cell types have different responses to sEV-derived miRs, perhaps because of differences in tetraspanin composition. Therefore, the authors detail an unexpected role for miR-574-5p in adenocarcinoma. Recipient cells have their PGE2-biosynthesis suppressed due to the action of miR-574-5p, secreted by the sEV [135]. Human lung tumor development is promoted by mPGES-1 induction when miR-574-5p acts as an RNA decoy for CUGBP1. By downregulating PTPRU expression, miR-574-5p increases tyrosine phosphorylation of β-catenin and stimulates NSCLC cell motility and invasion [136].

MiR-378 was shown to be overexpressed in highly invasive A549 and SK-LU-1 (human lung adenocarcinoma cell line) NSCLC sub-cell lines, whereas miR-1827 was found to be under-expressed. In addition, investigators uncovered how they play a role in regulating cell invasion, migration, and angiogenesis [137]. In vitro, primary lung adenocarcinoma cells were inhibited by miR-1827 when MYC or FAM83F overexpression was present. Furthermore, miR-1827 reduced tumor development by decreasing MYC and FAM83F levels in in vivo tests. Researchers conclude that miR-1827 inhibits lung cancer progression by targeting MYC and FAM83F, two oncogenic genes [138] (Table 3). Under specific settings, miRNAs may act as oncogenes or tumor suppressors. Cancer hallmarks such as proliferation signaling, evasion of growth suppressors, resistance to cell death, invasion, metastasis, and angiogenesis have all been demonstrated to be influenced by miRNA dysregulation. Evidence is mounting that miRNAs in lung cancer have a role as tumor suppressors or oncomirs by regulating the expression of target mRNA to impact biological processes central to tumorigenesis, including cell proliferation, invasion, angiogenesis, and immune evasion. Understanding the functions of the several microRNAs that have a role in various lung cancer subtypes is essential for advancing therapeutic and diagnostic approaches. The following discussion will focus on the function of several microRNAs in lung cancer.

**Table 3:** The function of microRNAs in lung cancer (SCLC and NSCLC). miRNAs can regulate lung cancer cells by targeting various genes. This regulation of miRNAs includes upregulation as well as downregulation

miRNAs	Target genes	Type of lung cancer	Explain	Ref.
let-7 family	K-RAS, CDC25A, CDK6, cyclin D, LIN28, MYC, HMGA2, HOXA9, TGFBR1, BCL-2, MAP4K3, FUS1	NSCLC	A decrease of let-7 in cancer cells leads to accelerated tumor growth, and the let-7 expression amount in serum shows cancer cell development.	[139, 140]
miR-15 family	PEBP4, TRAIL	NSCLC	miR-15 family was able to suppress cell development and stimulate apoptosis.	[141]
miR-25	Cyclin E2, CDK2	NSCLC/SCLC	miR-25 downregulation effect on SCLC growth and overexpression in NSCLC.	[142, 143]
miR-27a	Sprouty2, EGFR, c-MET	SCLC	MiR-27a was continuously reduced in sphere-creating cells.	[142]
miR-31	TSP-1, LATS2, PPP2R2A	NSCLC	A decrease in miR-31 expression increases lung cancer cell proliferation and migration.	[113, 142]
miR-34 family	MET, MYC, BCL2, E2F3, SIRT1/p53	NSCLC	miR-34b has low concentrations in NSCLC tissues.	[142]
miR-17-92 cluster	HIF-1a, PTEN, BCL2L11, CDKNA, RB	NSCLC/SCLC	miR-17-92 is upregulated in lung tumors and augments the number of cancer cells.	[142]
miR-126	Crk, VEGFA, SLC7A5	SCLC	Upregulation of miR-126 has a negative influence on SCLC cell proliferation and development.	[142, 144]
miR-130	PTEN	NSCLC	miR-130 has a tumor inhibitory effect on the tumorigenesis of NSCLC via targeting PTEN.	[145]
miR-138	H2AX	NSCLC/SCLC	miR-138 is considerably downregulated in SCLC tumors.	[142]
miR-155	Hexokinase 2, APAf-1, TCF4, TF	NSCLC	miR-155 levels are elevated in NSCLC, and upregulation of miR-155 considerably enhanced A549 cell development.	[139, 140, 142, 146]
miR-181	Bcl-2	NSCLC	miR-181 is a tumor suppressor that targets Bcl-2 expression.	[147]
miR-182	RGSIV	NSCLC	miR-182 is overexpressed in NSCLC and is an oncomiR to increase tumor cell proliferation.	[142]
miR-184	c-Myc	SCLC	miR-184, as a tumor suppressor, prevents cancer cell development and metastasis. Decreased miR-184 rate may forecast a worse prognosis in NSCLC patients.	[141]
miR-197	Fus1, p53	NSCLC	Increased miR-197 expression is associated with decreased Fus1 expression in NSCLC.	[148, 149]
miR-200 family	ZEB, E-cadherin, vimentin, Foxf2, NFkB, ETAR, SMAD3	NSCLC	miR-200 family by targeting oncogenes led to increased metastasis in NSCLC.	[141, 142]
miR-494	IGF2BP1, PTEN	NSCLC	Increased miR-494 expression is related to increased proliferation, decreased differentiation of tumor tissues, and increased feasibility of initial invasion and metastasis.	[139, 142]
miR-520a-3p	HOXD8, MAP3K2	NSCLC/SCLC	miR-520a-3p can induce apoptosis of cancer cells and suppress tumor stem cell phenotype in NSCLC and SCLC.	[150]
miR-574-5p	PTPRU, EPAS1	SCLC	miR-574-5p improved considerably and decreased the metastasis of SCLC.	[151]
miR-1827	RBX1, CRKL	SCLC	miR-1827 regulates lung cancer cell invasion and migration by EMT.	[137]
miR-21	EGFR, PTEN	NSCLC	miR-21 post-transcriptionally downregulates the expression of tumor suppressor PTEN and induces growth and invasion in NSCLC.	[152]

### Mir-17-92 cluster

miR-17-92 cluster (oncomiR-1) has varying effects on the pathogenesis of lung cancer. Overexpression of members of this miRNA (miR-17, miR-18a, miR-19a, miR-19b, miR-20a, and miR-92a) affects the course of lung tumors. The synergistic efficacy of miR-92a and miR-18a on NSCLC and anti-miR-18a therapy inhibited the tumor development level of xenografted mice remarkably. In addition, this miRNA with special gene augmentation may function in the SCLC [153]. Moreover, the novel investigation has shown that Docosahexaenoic acid (DHA), as a new curative, regulates the expression miR-17-92 and suppresses cell migration and survival of cancer cells in lung malignancy [154]. Sprouty 4 (SPRY4) was a direct target of miR-92a, and its overexpression prevented the intensification of NSCLC stimulated through miR-92a. Moreover, the tumor xenograft method demonstrated that miR-92a facilitated tumor growth by suppressing the expression of SPRY4 and interceding Epithelial-Mesenchymal Transition (EMT) in vivo. In addition, researchers investigated the synergistic efficacy of miR-92a and miR-18a on NSCLC and found that antagomiR-18a treatment inhibited the tumor development level of xenografted mice remarkably [153].

### miR-31

The expression of miR-31 was elevated in lung cancer, among other malignancies. When comparing lung adenocarcinoma samples with lymph node metastases to those without, miR-31 expression was higher in the latter. Additionally, miR-31 activated the ERK1/2 signaling pathway, which promoted the migratory, invasive, and proliferative capabilities of NSCLC cells. Moreover, miR-31 promoted the mutant KRAS-mediated lung carcinogenesis by inhibiting the RAS/MAPK negative regulator [155]. Oncogenic miR-31 can contribute to chemoresistance in lung cancer treatment [156]. Moreover, more excellent rates of circulating exosomal miR-31-5p were identified in LUAD patients, particularly in the metastatic stage of lung cancer LUAD. This miRNA remarkably augments the triggering of MEK/ERK signaling, as a result of that implicated in cancer development both in vitro and in vivo [157]. Furthermore, oncogenic efficacy miR-31-5p assisted LUAD cell development interceded via the TNS1/p53 axis [158]. RNA-binding protein Quaking-5 (QKI-5), a main isoform of QKIs, suppresses tumor development in NSCLC. miR-31/QKI-5/p21-CDK4–CDK6 axis might have essential roles in developing NSCLC, and targeting this axis can be used as a therapeutic capability method for NSCLC [155].

### miR-200 family

All members of the miR-200 family possess potent suppressive effects on cell differentiation, cancer cell increase, tumor development, and metastasis. These miRNAs target genes that assist metastasis in the onset of NSCLC. Constant expression of cluster I miR-200s decreased protein and mRNA rates of the vascular endothelial growth factor receptor 1 (VEGFR1) protein in LUAD 344SQ cells. MiR-200 family has a therapeutic and diagnostic role in lung cancer [159, 160]. In another study, researchers showed that these miRNAs are potent inhibitors for lung cancer LUAD metastasis. miR-200 expression is remarkably repressed in mouse metastatic lung cancer LUAD, and miR-200 reduction powerfully relates to weak patient viability [161]. EMT is an essential process involved in the integrity of organogenesis and tissue differentiation also tissue regeneration, organ fibrosis, and the development of cancer. Multiple miRNAs were offered to form the network controlling EMT in lung cancer, among which miR-200 family members play essential functions in the inhibition of EMT [162]. In a study, researchers recognized Foxf2 as a common, new, and direct target of the miR-200 family. Foxf2 expression tightly relates to the transcription factor Zeb1 and increases in mesenchymal-like metastatic lung cancer cells. Foxf2 expression stimulated strong EMT, migration, invasion, and metastasis in lung cancer cells, whereas Foxf2 suppression remarkably inhibited these phenotypes. Researchers also showed that Foxf2 transcriptionally inhibits E-cadherin and miR-200, independent of Zeb1, to create a double-negative feedback loop [163].

### Let-7 family

The let-7 family is downregulated in NSCLC. A decrease of let-7 expression in tumor cells will result in rapid tumor development derived from disturbances to the signaling networks, including Ras GTPase. A negative feedback loop in which let-7a binds to the 3ʹ UTR of the DICER1 gene has been detected in lung cancer [164, 165]. Body fluids let-7 expression amount could dependably show the proliferation of the tumor tissue in persons with lung tumors. Reduced let-7a levels imply unfavorable postoperative prognosis in patients with NSCLC [166]. Let-7 is a tumor suppressor microRNA targeting the KRAS lung oncogene. Let-7a downregulation is reversible during the early stages of lung carcinogenesis but is irreversible in cancer cells. Tumor cell growth, invasion, and migration are slowed by let-7a transfection into LUAD cells. DNA methylation is one of the mechanisms that control let-7a expression. Let-7a-promoter hypomethylation increases the expression of let-7a, thus decreasing the development of LUAD cells. Let-7a is downregulated in response to hypermethylation in epithelial cancers, and this downregulation is linked with poor prognosis [167, 168]. Epidermal EGFR‑TKI resistance shows a main barrier in the therapy of NSCLC. In an investigation, it was detected that let‑7 family expression was downregulated, and miR‑17 family expression was upregulated in gefitinib‑resistant PC9/GR cells compared with gefitinib‑sensitive PC9 cells. The downregulation of let‑7 and upregulation of miR‑17 have considerable clinical relation to gefitinib resistance in NSCLC. In addition, it was demonstrated that the downregulation of let‑7 and upregulation of miR‑17 increased resistance to gefitinib by controlling the self‑renewal potential of NSCLC cells. Moreover, let‑7 is involved in maintaining stem cell preservation by regulating the target gene MYC. In NSCLC cells, down expression of let‑7 enhanced MYC expression to assist in preserving the undifferentiated situation. Therefore, let‑7 increased self‑renewal, typical of stem cell‑like attributes, and led to gefitinib resistance [169].

### miR-101

In another study, miRNA-101 controls the myeloid cell leukemia-1 (Mcl-1) gene as a target gene. The decreased level of miRNA-101 is related to elevated levels of Mcl-1, and it caused lung cancer survival increase. The outcomes of this research displayed that the expression of Mcl-1 is repressed by miRNA-101 and decreased the development of A549 cells. The sensitivity of the A549 cells to etoposide was synergistically enhanced by the transfection of miRNA-101. Apoptosis assay demonstrated that apoptosis augmented the etoposide-mediated apoptosis performed by miRNA-101. Cell proliferation and survival were inhibited by the upregulation of miRNA-101 and suppressing Mcl-1 expression sensitized A549 cells to etoposide, and it verified miRNA-101 as an efficient therapeutic strategy in NSCLC [170]. Overexpression of miR-101-5p repressed the development of NSCLC via activation CXCL6 and might be a robust curative target for NSCLC [171]. miR‑101‑3p upregulation sensitized LUAD cells to ionizing radiation through reducing the capabilities of LUAD cell proliferation, colony development, DNA damage repair, and enhancing caspase‑3 acting and apoptosis of LUAD cells next ionizing radiation. Moreover, based on bioinformatics study and luciferase method, baculoviral IAP repeat containing 5 (BIRC5) was recognized as a direct target of miR‑101‑3p. Enhanced BIRC5 expression reversed the miR‑101‑3p‑interceded augment in LUAD cell radiotherapy sensibility [172]. In addition, this miRNA inhibited NSCLC development and metastasis by targeting ZEB1 [173]. In addition, the mouse xenograft study demonstrated that cancer-related fibroblasts were associated with tumor development, whereas miR-101-3p decreased cancer-related fibroblasts’ effect. These findings showed a new mechanism that cancer-related fibroblasts facilitated lung cancer metastasis capability through miR-101-3p/VEGFA/AKT signaling pathway, offering miR-101-3p as a potential candidate for metastasis therapy [174].

### miR-106a

In another research, scientists studied miR-106a in lung malignancy patients with bone metastasis (BM). They demonstrated that the expression of miR-106a stimulates bone metastasis of lung LUAD. Upregulating miR-106a is binding to tumor protein 53-induced nuclear protein 1 (TP53INP1), and it promotes metastasis development, containing EMT, cell migration, and autophagy-dependent death. Scientists demonstrated that by silencing miR-106a and promoting the expression of TP53INP1, it would have a therapeutic effect in lung cancer patients with BM by regulating autophagy-dependent cell death, EMT, and cell migration. Restoring the expression of TP53INP1 through suppressing miR-106a may be a new therapeutic method for bone metastasis in LUAD [175]. In addition, miR-106a suppressed the development and metastasis of NSCLC cells by reducing PTEN expression [123].

### miR-15 family

The miR-15 family is capable of suppressing cancer cell development and also the induction of cancer cell death [176, 177]. In another study, investigators showed that miR-195-5p might have decreased the chemotherapy resistance to cisplatinum in the A549/DDP cells and reduced NSCLCs migration and invasion. Increased expression of FGF2 led to increased cisplatinum resistance in the lung tumor cells, while miR-195-5p might decrease chemotherapy resistance [178]. Researchers have shown that miR-15/16 family members are tumor suppressors in NSCLC. The MesomiR 1 investigation is a first-in-human Phase I trial of experimental treatment with miR-15/16 mimics encapsulated in EnGeneIC Delivery Vehicle (EDV) TM nanoparticles (NPs) targeted with EGFR antibodies (TargomiRs). 3/5 patients recovered quality of life parameters during the therapy period [179]. In addition, suppression of proliferation, migration, and invasion of NSCLC cells can be obtained with targeted regulation of Smad3 through miR‑15a [124].

### miR-34 family

miR-34a is a part of the p53 tumor suppressor network and downregulates this miRNA, leading to the progression of NSCLC. MiR-34a directly targets the EGFR. This behavior can also be seen in the expression of miR-34b as it has been lowered in NSCLC [180]. Moreover, the miR-34 family was reduced in occupational-exposure-associated lung LUSC patients. The miR-34b/c function is more than miR-34a in suppressing LUSC development, spreading, and metastasis by targeting the Notch1 pathway. Expression of miR-34a was reversely related to CD44 expression in vivo [181]. In vitro methods showed that miR-34a and miR-34b/c suppresses LUSC cells proliferation, migration, and invasion by the Notch1 pathway, while miR-34b/c efficacy more than miR-34a does. As miR-34a was remarkably reduced in cancer recurrence, the further study of relevance between miR-34a and stem cell adhesion molecular CD44 demonstrated that miR-34a was significantly related to CD44 rates in patients. Knockdown of CD44 markedly inhibited miR-34a interceded prevention of cell migration and invasion. Treating the purified CD44^hi^ cells with miR-34 overexpression lentivirus suppressed the tumor development. By contrast, anti-miR-34 facilitated tumor progression of CD44^low^ cells. miR-34 family offers a theoretical basis for LUSC treatment and a biomarker candidate for LUSC prognosis [181]. MiR-34a is a great tumor suppressor that may be used in cancer treatment. Numerous investigations on miR-34a therapies have been conducted, and the importance of this compound in suppressing tumors in cancer has been confirmed. Nevertheless, miR-34a is another intriguing treatment option for cancer. Therefore, a great deal of research on the role, adverse effects, and target treatment of miR-34 in lung cancer has to be done.

### miR-184 and miR-574-5p

miR-184 appreciably reduced the metastasis of SCLC, while miR-574–5p increased in SCLC. These miRNAs were detected to get involved in β-catenin signaling via inhibiting protein tyrosine phosphatase receptor type, U (Ptpru) or hypoxia-inducible factor-2alpha. Moreover, miR-574–5p was substantiated as an autonomous predictive danger agent for SCLC. MiR-574-5p expression increased in LS-SCLC compared with ES-SCLC, while miR-184 downregulated [151, 182]. Endothelial PAS domain protein 1 (EPAS1), as well as recognized as hypoxia-inducible factor 2 alpha subunit (HIF-2α), is a kind of transcription agent that firstly stimulates the transcriptional reaction to hypoxia. Multiple lines of proof have demonstrated that EPAS1 is associated with several perspectives of cancers, such as cell proliferation, angiogenesis, apoptosis, metabolism, metastasis, and resistance to chemotherapy. miR-184 and miR-574-5p are involved in β-catenin signaling by repressing protein tyrosine phosphatase receptor type U (PTPRU) or EPAS1. Clinically, researchers established that up-expression of miR-574–5p is related to reduced progression-free survival and overall survival in SCLC patients. miR-574–5p and miR-184 are implicated in SCLC migration and invasion activities, potentially serving as novel prognostic agents and clinical therapeutic targets for SCLC [183]. HOXA transcript at the distal tip (HOTTIP)‑knockdown may result in a considerable enhancement in E‑cadherin expression and a reduction in vimentin (VIM) expression; these proteins are two crucial markers of EMT. In a study, researchers showed that HOTTIP may be implicated in the EMT of SCLC through binding to miR‑574‑5p and that miR‑574‑5p may act by VIM, which is a crucial marker of EMT [184].

### miR-1827

The functions miR-1827 include modulated cancer invasion, migration, and angiogenesis in LUAD via binding to RBX1 and CRKL. MiR-1827 mimics had a decreased rate of metastases and ectopic vessels [185]. Caveolin-1 (CAV-1) is directly targeted through miR-1827. Restoration of CAV-1 remarkably decreased miR-1827’s effect on anoikis resistance in A549 cells. A new signaling axis of miR-1827/CAV-1 in controlling anoikis resistance may be used as a potential therapeutic agent for metastatic NSCLC [186]. In a different study, researchers discovered that miR-1827 expression was downregulated in LUAD and that this low expression was strongly connected with both the disease’s development and the patients’ bad prognosis. In vitro, miR-1827 overexpression significantly decreased the malignancy of primary LUAD cells. In LUAD cells, miR-1827 was shown to target two genes: MYC and FAM83F. These two genes had elevated levels of LUAD, and a strong correlation was shown between their high expression and the disease’s advancement, and the unfavorable prognosis of affected individuals. The effects of miR-1827 on primary lung adenocarcinoma cells in vitro were reduced by overexpressing MYC or FAM83F. Furthermore, miR-1827 was shown to prevent tumor development by lowering MYC and FAM83F levels in in vivo tests. In conclusion, miR-1827 may inhibit the growth of LUAD by focusing on the cancer-causing genes FAM83F and MYC [138].

Many of these miRNAs function as tumor suppressors or oncogene repressors in lung cancer, respectively, and hence contribute to or repress the cancer phenotype. The function of tumor suppressor and oncogene miRNAs will be explored below.

## Tumor-suppressor or oncogenes miRNAs in NSCLC

Significant miRNAs have been detected restrained in malignancies compared to similar healthy tissues as a sign of the typical loss of separation of cancer cells. miRNAs are upregulated and downregulated in cancer tissues compared to the healthy tissues, and they can be interested as oncogenes or tumor-suppressors [187]. miRNAs have different roles in NSCLC, such as tumor-suppressor miRNAs (such as let-7, miR-34 family, miR-200 family, miR-451, miR-29 family, miR-126, miR-101, miR-192, and miR-545), and oncogenic miRNAs (e.g., miR-17-92 cluster, miR-221, miR-222, miR-21, and miR-31) [88] (Table 4). For example, the let-7 family is downregulated in NSCLC. Failure of let-7 expression in tumor cells will result in accelerated cancer progress, which is derived from disturbances to the signaling networks, including the Ras GTPase. A downregulated in let-7a binds to the 3ʹ UTR of DICER and has been identified in NSCLC [164, 188]. MiR-34a downregulated in NSCLC via a direct effect on EGFR expression, and miR-34b levels in tumor tissues were lesser than doubtful lung malignancy cases [189]. OncomiR-1 could per se target the tumor suppressor PTEN. Overexpression of the miR-17-92 cluster affects the course of lung tumors [88, 190]. In another study, scientists investigated the efficiency of miR-320b on angiogenesis and tumor development in lung malignancy. Results displayed that the expression rate of miR-320b is low in tumor tissues. HNF4G was recognized as a target for miR-320b. HNF4G showed high expression in lung malignancy [191]. In another study, investigators displayed that the expression rate of miR-320a is reduced in NSCLC. However, ZEB1 expression was high. Similarly, metastasis, proliferation, and metastasis of NSCLC were reduced by upregulation of miR-320a, and the apoptosis process was promoted. The expression of RAD51AP1 by ZEB1 caused cancer development in vivo. As a result, ZEB1 suppressed the expression of miR-320a, and it caused up-regulation of RAD51AP1 and stimulated metastasis in NSCLC [192]. miR-506 is proven as a tumor suppressor in NSCLC. This miRNA can prevent NSCLC development by regulating TULP3. This investigation showed that the expression rate of miR-506 is high in NSCLC cell lines, and it inhibited cancer cell development and promoted programmed cell death in H1299 and A549 cells. Due to the upregulation of miR-506, the expression rate of pro-apoptotic proteins augmented; however, the expression of anti-apoptotic proteins was reduced. In conclusion, miR-506 has a regulatory effect on TULP3 and inhibits the development of NSCLC [193]. In lung cancer, miRNAs act as tumor suppressors or oncogenes related to the targets. Alterations in miRNA expression rate are associated with tumor beginning, development, and metastasis. MiRNAs can control gene expression and, therefore, influence the activity situation of various signaling pathways, such as AKT, JAK-STAT, MAPK, TGF-β, WNT, and ERK signaling pathways [194]. Here, we focus on many cases where a single miRNA has a dual role in lung cancer as a tumor suppressor and an oncogene. Learning how microRNAs may either promote tumor growth or inhibit it is crucial for developing miRNA-based therapies for lung cancer. We urge against jumping into this without first gaining a solid grounding on miRNAs from a holistic perspective that considers the considerations as mentioned above. Therefore, we suggest that preclinical investigations of putative miRNA therapies use immunocompetent mice models, which more accurately represent the tumor microenvironment. It would also be beneficial to further research how miRNAs interact with currently used treatments.

We will discuss the usage of miRNA mimics and anti-miRNAs in the treatment of lung cancer LC in the next part.

**Table 4:** Tumor-suppressor and oncogenes miRNAs in NSCLC

miRNAs	miRNAs action	Effect on the expression of their target genes.	Function^**^	Target genes
Let-7	Tumor suppresser	Downregulated	A/B/C/D/E	K-RAS, MYC, HMGA2,
miR-15a	Tumor suppresser	Downregulated	A/B	CDK6, CDC25, CyclinD1/2
miR-16/1	Tumor suppresser	Downregulated	A/B	CDK6, CDC25, CyclinD1/2
miR-21	Oncogenes	Downregulated	A/B/C/D/E	PTEN, BTG2, PDCD4, FASLG, RHOB
miR-29	Tumor suppresser	Downregulated	A/B/C	P85α, DNMTs, MCL1, P53
miR-31	Oncogenes	Downregulated	A/B	LATS2, PPP2R2A, PP2A
miR-34 family	Tumor suppresser	Downregulated	A/B/D/E	E2F1, AXL, SNAIL1
miR-17-92 cluster	Oncogenes	Upregulated	C/D/E/I	PTEN, HIF-1α
miR-101	Tumor suppresser	Downregulated	C	MLC1, EZH2
miR-126	Tumor suppresser	Downregulated	A/B/I	EGFL7, VEGF-A
miR-192	Tumor suppresser	Downregulated	A/B	RB1
miR-200 family	Tumor suppresser	Upregulated	A/B/D/E/F/I	ZEB, E-cadherin, KDR, Foxf2, NFkB, ETAR, SMAD3
miR-212	Tumor suppresser	Downregulated	A/B	PED, PTCH1
miR-218	Tumor suppresser	Downregulated	D/E	PXN
miR-221	Oncogenes	Upregulated	C/D/E	PTEN, BIM, PUMA, TIMP-2
miR-222	Oncogenes	Upregulated	C/D/E	PTEN, BIM, PUMA, TIMP-2
miR-301	Oncogenes	Upregulated	A/B	SKA2, ERK
miR-449 family	Tumor suppresser	Downregulated	A/B	E2F1, p53, HDACs
miR-451	Tumor suppresser	Downregulated	C	RAB14, Bax, Bad
miR-494	Oncogenes	Downregulated	C/D/E	PTEN
miR-545	Tumor suppresser	Downregulated	A/B	cyclin D1, CDK4

**Different functions of miRNA in NSCLC, including (A) cell proliferation, (B) cell-division cycle, (C) apoptosis, (D) EMT, (E) metastasis, (F) anti-cancer stem cell (CSC), (I) angiogenesis

## miRNAs mimic and anti-miRNAs in Lung cancer therapy

The progress of novel therapy methods has significantly increased life expectancy by about five years and decreased overall fatality numbers. In contrast, the increasing categorization of malignancies has multiplied, and the diversity and specialization of therapeutic methods have retarded behind. The pleiotropic character of miRNAs to target hundreds of genes makes them a potential objective for tumor treatment investigations. miRNAs have minor toxicity and lesser immune reaction than other novel therapy methods, such as DNA or protein-base therapy [195]. According to up/down-regulation of miRNAs, the treatment function of miRNAs includes two strategies: 1- Barricade oncogenic miRNAs via applying miRNA antagonists, including anti-miRs, locked-nucleic acids (LNA) or antagomiRs, and 2- miRNA substitution strategy, includes the re-presentation of a tumor suppressor miRNA mimic to bring back the damage of performance. miRNA antagonists are ss-RNA (about 21–23 nt), which act through supplementary base matching with miRNAs [196]. AntimiRs comprising cholesterol, interlaced through a 2′ -O-methyl bond, recognized as the antigomiRs, are supplementary to the mature miRNA sequence and include various phosphorothioate moieties to improve resistance. The miRNA antagonist chemical structure features presently in improvement usage unconjugated phosphorothioate oligonucleotides with numerous additional great-affinity 2′ sugar alterations, including 2′ Omethoxyethyl (2′ MOE) or LNA, which contain the maximum affinity [197]. MiRNA mimetics show a further rate of complication compared with anti-miRs. This treatment method can cause adverse events when new miRNAs are presented in a cell. However, these presumptions are comprehensible; in vivo, observations of toxicity caused by miRNA mimics do not exist yet. Animals experiments that rate the treatment transfer of tumor suppressor miRNAs were unsuccessful in diagnosing negative phenomena dependent on miRNAs and offer that the transfer of miRNA to healthy tissues was excellently tolerated [198]. For example, in vitro experiments on NSCLC cell culture showed that the hsa-miR-125a-3p and − 5p are downregulated in this type of lung malignancy and exhibit distinct impacts on the migration and invasion of NSCLC. These miRNAs are directly delivered as miRNA mimetics into A549 [199]. Phong Trang et al. developed the systemic transport of miR-34a mimics using a neutral lipid emulsion (NLE) in mice. This delivery strategy causes aggregation of miR-34a in cancer tissues, suppression of miR-34a targets, and strong suppression of NSCLC in mice [200] (Table 5). Effective therapy for lung cancer should affect the necessary controlling and coding sequences associated with the pathogenesis of lung cancer [201]. MiRNA mimics and anti-miRNAs, which reinstate miRNA expression or inhibit aberrantly expressed miRNAs, respectively, are remarkably regarded therapeutic methods for successfully manipulating miRNA levels. Thus, miRNA delivery systems that simplify the proficient and safe transfer of miRNA-based therapeutics are essential to the clinical success of these pharmaceuticals [81]. One method for determining the targets and functions of specific miRNAs is transfection with miRNA mimics or inhibitors. After transfected into a cell, synthetic miRNA mimics function similarly to endogenous miRNAs. Compared to protein-based medications and even plasmid-DNA based gene therapy, miRNAs have fewer adverse effects and are less immunogenic.

antimiR oligonucleotides must be optimized for higher binding affinity, greater nuclease resistance, and more effective in vivo delivery to inhibit miRNA effectively. As we’ll see below, targeted therapy is crucial for minimizing adverse effects and maximizing microRNAs’ durability and efficacy.

## miRNA delivery methods in Lung cancer

**Fig. 4:**
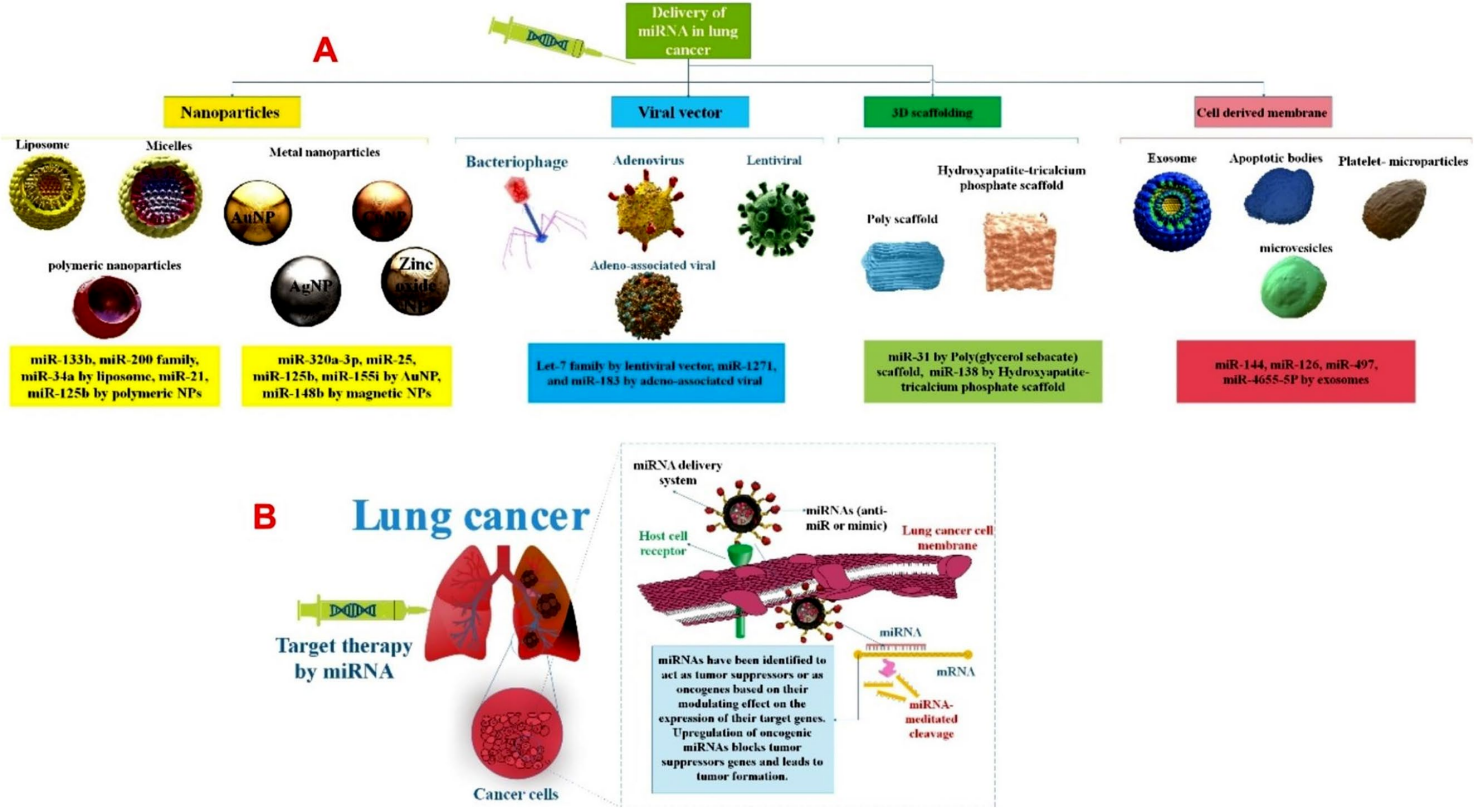
(**A**) Differential miRNAs encapsulated in diverse carriers for lung cancer treatment, including viral vectors, cell-derived membranes, NPs, and three-dimensional scaffolding. (**B**) tailored miRNA delivery for lung cancer using a unique carrier-based approach

miRNAs without special carriers or uncovered miRNAs are hard to enter into cells and decompose quickly in exterior and interior surroundings [202]. Various miRNA delivery methods have been generated to solve this problem. Moreover, targeted miRNA delivery has displayed in vivo and in vitro hopeful outcomes with curative effectiveness [203]. The clinicians use multiple delivery strategies for miRNA, including both local and systemic. For the miRNA delivery, viruses, NPs (e.g., different lipid-based nanoparticles (LNPs), niosomes, polymeric NPs, inorganic NPs, gold NPs, scaffolds, etc.), and extracellular vesicles such as exosomes are used [52, 204–207]. In addition, local miRNA delivery is being done either with or without the carriers. This strategy came with less toxicity as opposed to systemic delivery. Systemic delivery includes lipid-based, polymer-based, exosomes, scaffold-based, and viral vectors [208–211]. Also, there is a class of miRNA antagonists or miRNA mimics, which are poly-anionic molecules with fewer molecular weights. These molecules are highly soluble in water and appropriate for intravenous and subcutaneous administrations. The pharmacological endurance effect of miRNA antagonists or mimics depends upon their retention in the target tissue. Following systemic injection, the drop in the number of miRNA antagonists or mimics in the lung tissue is steep. Various viral vectors can transfect most cell kinds in vitro and in vivo. Also, these viral vectors are insertional mutagenesis, and safety matters are significant obstacles to clinical translation [45, 212, 213] (Fig. 4). For example, in NSCLC lung cancer, viral vectors were used to deliver the let-7 family of miRNA [118]. LNPs as miRNA delivery systems are one of the most frequently used in tumor cell culture. This was done via siPORT NeoFX, a commercially accessible LNP that transfected lipoplexes pre-miR-133b in A549 NSCLC. In NSCLC, miR-34a and Let-7 family are delivered via NLEs. NLEs are epicene load NPs with slight side effects and less delivery toxicity to tumor sites. NPs aid the delivery of miRNAs by providing physical constancy to the unstable miRNA construction and preserving the miRNA from nuclease destruction, as well as supporting the effective silencing of target genes. For example, miR-34 mimic (MRX34) delivered in lipid NPs to human studies in the invasion Kras, Trp53 NSCLC animal model resulted in considerable tumor decrease. The scaffold-based delivery proposal is a three-dimensional (3D) delivery of miRNA at target tissue that includes entrapping or immobilizing the miRNA into the tissue engineering scaffold addresses [214–217] (Table 5). Between RNA molecules, miRNAs show an encouraging solution versus cancer. Despite the capability to target several pathways, miRNA-based therapeutics struggle to attain phase 3 clinical study. A cause of this delay is related to the side effects of selective administration of miRNAs to their target, the tumor cell. For this reason, an effective delivery system is vital. In this regard, using NPs and cell derivatives such as exosomes can help in the future of this treatment method [218–220]. Large-scale production, high loading capacity, stability, extended half-life in circulation, minimum toxicity, and prevention of fast degradation of their payload should all be significant research foci for miRNA delivery techniques. The following sections will discuss how miRNAs, in addition to the previously mentioned roles, also play a role in the drug resistance of lung tumor cells by targeting genes related to drug resistance or affecting genes related to lung cell proliferation in lung cancer.

**Table 5:** Summary of studies using miRNAs mimics and anti-miRNAs in drug delivery systems for lung cancer therapy

Delivery Vehicle	Targeted miRNA	Antagonist / Mimics	Type of lung cancer	Effect	Ref
Sense and antisense 2’-O-methyl oligonucleotides	miR-125-3p/5p	Mimics	NSCLC	Inhabited migration and invasion of NSCLC	[221]
Lentiviral vector	Let-7	Mimics	NSCLC	This tumor suppressor miRNA inhibits the growth of cultured lung cancer cells	[118, 222]
LPH-PEG-GC4	miR-34a	Mimics	SCLC/ NSCLC	Cause of programmed cell death and inhibited XXXurviving expression and downregulated MAPK pathway in B16F10 cells.	[223, 224]
pMSCV-Pur vector or directly	miR-1254	Antagonist	NSCLC	miR-1254 inhibition caused suppresses lung cancer cell proliferation.	[225]
Cationic DOTMA lipoplexes	miR-29b	Mimics	NSCLC	Decreased expression of the main target oncogenes and suppression of cancer development.	[226]
Lenti-miR vector or directly	miR-224	Antagonist	NSCLC	Upregulated miR-224 expression promotes cell migration, invasion, and proliferation	[227]
Sense/antisense	has-miR-125a-3p	Antagonist	NSCLC	Overexpression of this miRNA effectively suppresses cell development and invasion via modulating the mouse double minute 2 homolog/ p53 signaling pathway.	[228]
NLE	miR-34a/ let-7	Mimics	NSCLC	Suppression of tumor cell proliferation and generation of apoptosis.	[229, 230]

## miRNAs in Lung cancer medication-resistant

Several treatment techniques, separately or combined, including chemotherapy, radiotherapy, surgery, targeted treatments, cell therapy, and immunotherapy, are utilized in lung cancer [231, 232]. About 25–30% of NSCLC and 2–6% of SCLC are cured via surgery. In SCLC, surgery is performed with chemotherapy and radiation therapy to recover the patient [233, 234]. Platinum, composed of paclitaxel, docetaxel, gemcitabine, vinorelbine, and irinotecan, is suggested as a foundation alternative for most patients with an aggressive form of lung cancer that has spread extensively [235–238]. Moreover, miRNAs could control the expression of essential genes involved in medication resistance, including regulation of cell development, DNA modification, and programmed cell death [239–241]. Moreover, lung cancer cells may obtain cancer chemotherapy resistance through modifying DNA damage repair methods, including general recombination (GR), which has been displayed to be regulated via miRNAs. It has newly been shown that miR-7-5p interceded inhibition of the Poly (ADP-ribose) polymerase 1 (PARP 1) could interfere with the GR regeneration pathway stimulated via doxorubicin in doxorubicin-resistant SCLC cells and re-sensitivity to chemotherapeutic agents [242]. In another investigation, researchers showed that miR-194-5p enhanced the sensibility of NSCLC cells to doxorubicin via per se suppressing HIF-1. HIF-1 was enhanced in hypoxia-stimulated medication-resistant NSCLC cells [243]. Several miRNAs, such as miR-185–3p and miR-133a-3p, have been implicated in modulating the medication resistance of lung cancer patients via regulating EGFR signaling pathways [244, 245]. In another study, investigators showed that miR‑139‑5p increased cisplatin sensitivity in NSCLC cells by suppressing cell development and increasing programmed cell death by regulating Homeobox protein Hox‑B2 (HOXB2) [246]. MiR-181b was remarkably downregulated in cisplatin-resistant H446 SCLCs. Overexpression of miR-181b inhibited cancer cell development and metastasis in cisplatin-resistant H446 cells [247]. Beclin-1 modulated via miR-30a-5p plays a notable significance in the medication resistance of SCLC. Suppression of Beclin-1 via stimulation of miR-30a-5p may enhance the curative result through re-sensitization of the antineoplastic resistance in SCLC [248]. Upper tumor miR-92a-2* rates are related to chemoresistance and reduced survival rates in SCLC [249]. The correlation of Sirtuin- 1 (SIRT1) and miR-326 is studied in NSCLC. The expression levels of SIRT1 and miR-326 were measured in both H460 and chemoresistant cells H460-R. MiR-326 expression was reduced when SIRT1 expression was augmented in NSCLC. Chemotherapy-resistant NSCLC proliferation is inhibited by MiR-326 and increases apoptosis by suppressing SIRT1. As a result, SIRT1 expression is inhibited by miR-326 to inhibit HIF1α and elevate VEGFA due to decreased chemoresistance, thus inhibiting the progression of NSCLC [250, 251]. miRNAs not only play an essential function in initiation and development but also induce or suppress resistance to various therapeutic agents [252]. Recent research has shown that miRNAs target genes linked to drug resistance or affect genes involved in cell proliferation, cell cycle, and apoptosis, hence contributing to the drug resistance of tumor cells. MiRNAs often control the target genes of lung cancer to lessen chemotherapy resistance and halt the development of lung tumors.

Numerous studies suggested that miRNAs could be helpful tobiomarkers for lung cancer early detection. The function of miRNAs as lung cancer biomarkers has been discussed below.

## Circulating miRNA as a biomarker in Lung cancer

**Fig. 5:**
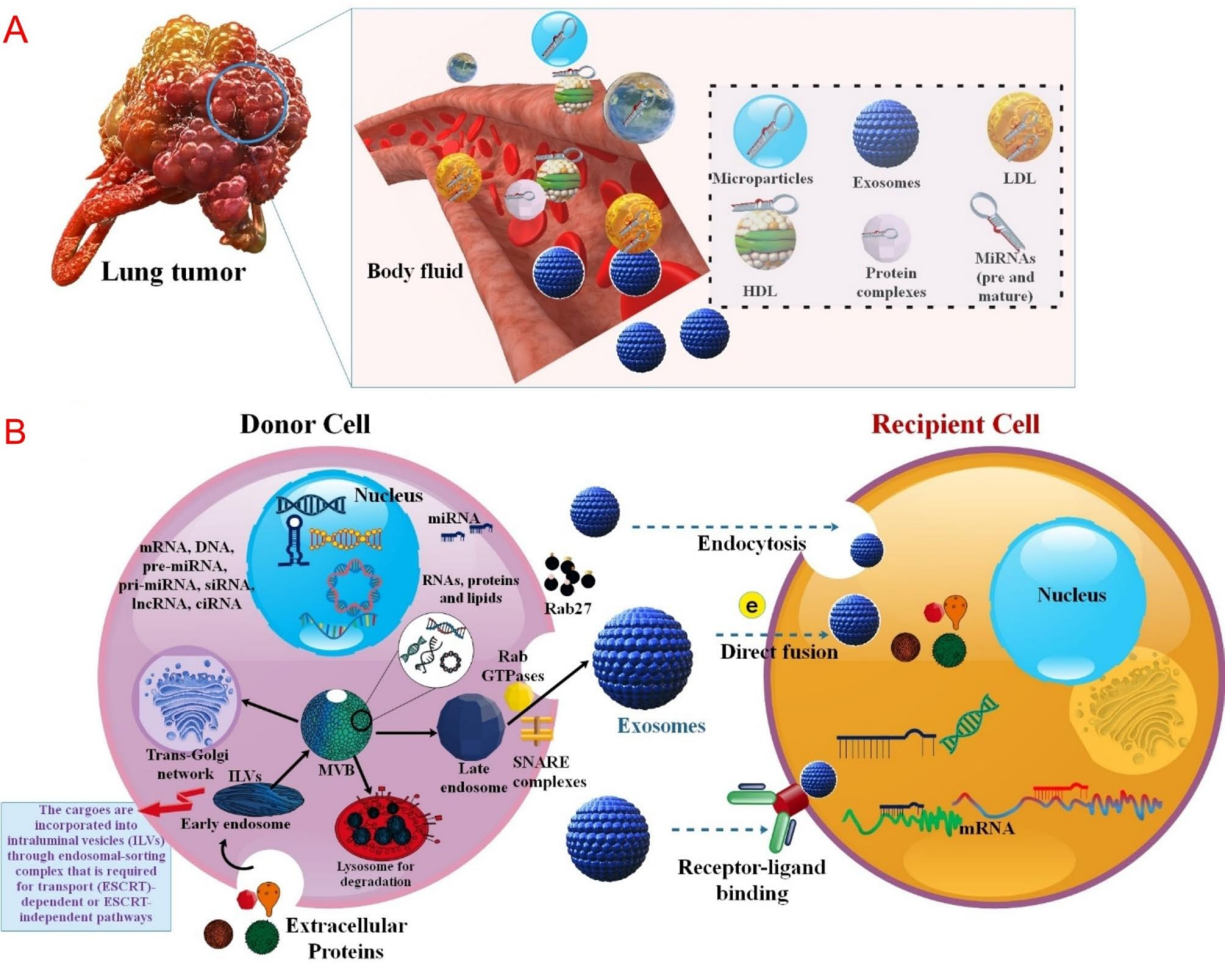
(**A**) Categorization of cell-free miRNAs and supposed origins of ci-miRNAs [254]. However, in different tissues involved in the ci-miRNA pool, most miRNAs may be obtained from blood. Ci-miRNAs can be released from necrotic or apoptotic cells, released vesicles, including microvesicles or EXOs in protein compounds and lipoprotein compounds (HDL and LDL compounds) [282, 283]. (**B**) EXO formation steps into donor cells and different uptake of EXOs in recipient cells [284]. EXOs fundamentally combine with the cell membrane either after Ca2+-related activation or behind the triggering of Rab-GTPases. Rab25 controls EXO connecting before and securing the cell membrane and Rab27b EXO discharge [285]

In 2008, the primary cell-free miRNA was reported in maternal plasma, and circulating miRNAs (ci-miRNA) were XXXs also described in blood serum. miRNAs can be distributed in numerous body fluids, including blood, urine, semen, saliva, milk, pleural effusions, and cerebrospinal fluid (CSF) [253, 254]. Ci-miRNA is a disease biomarker, and this miRNA helps detect cancers. Ci-miRNA is an excellent biomarker, including approachability in several bodily fluids, sequence protection among human and clinically significant animal models, and access to accurate and different detection methods [255]. Various sources for secretion circulating miRNAs, including those released by cells via shedding of microvesicles, exosomes, apoptotic bodies, HDLs, and protein complexes, also inactive released from cells [256]. Blood circulating miRNAs in two forms: cell-freely, related with Argonaut proteins, or in exosomes. Most circulating miRNAs are presented as binding with Ago2 [254]. Ci-miRNAs show a reassuring abeyant for the detection of lung cancer. The different expression levels of circulating miRNAs in the sample can be found at all stages, from primary to development and even afterward metastasis of malignancy, letting researchers detect the alterations in different conventional methods, such as qRT-PCR, microarray, and northern blotting [257]. For example, plasma miR-21-5p, miR-20a-5p, miR-141-3p, miR-145-5p, miR-155-5p, and miR-223-3p are appreciably augmented in NSCLC at steps I and II. Serum miRNAs, including miR-126-3p, miR-182-5p, miR-183-5p, and miR-210-3p, were also detected to contain primary diagnosis compared for NSCLC, displaying the same sensibility and quality as usual malignant tumor biomarker carcinoembryonic antigen (CEA). Moreover, pri-miR-944 and pri-miR-3662 could identify NSCLC at stages I–IIIA [258–260]. In plasma, exosomal rates of miR-361-5p and miR-181-5p were 10 times upper in LUAD than in LUSC. However, miR-320b was augmented in LUSC, and miR-9-5p was tremendously decreased in LUAD [261]. Lung cancer patients use Cisplatin (DDP) for its therapeutic effects; in a study, the regulatory impact of miR-106a-5p was evaluated for detecting DDP-resisting activity. Scientists have found that invasiveness, epithelial-mesenchymal transition (EMT), and migration are inhibited by MiR-106a-5p. However, apoptosis is enhanced in cisplatin (DDP)-resistant A549 (A549/DDP) cells via connecting JMJD6 per se, and it suggests MiR-106a-5p and JMJD6 as a potential biomarker in lung cancer [262] (Table 6). In another research, circ_0008717 is packaged stably into exosomes in NSCLC. Circ_0008717 increased regulation of P21-activated kinase 2 (PAK2) by utilizing miR-1287-5p, and it developed oncogenic effects in NSCLC. In conclusion, this study promotes exosomes circ_0008717 as a therapeutic agent for NSCLC patients, and it will also assist in understanding the mechanism of exosomal circRNA in biology [263] (Fig. 5). Moreover, in several studies, the diagnostic efficiency of circulating free and extracellular vesicle-related miRNAs in lung cancer was investigated. Due to the enhanced stability and widespread applicability, miRNA may be one promising biomarker for lung cancer detection [264]. Most of the requirements for an excellent biomarker, including accessibility, high specificity, and sensitivity, are met by these compounds. The study of miRNAs as biomarkers for different illnesses is still a fascinating area of study despite current constraints. Numerous studies have been conducted, and because of their increased accuracy and increased sample size, miRNAs will soon be employed in a larger and more clinical setting for the diagnosis and prognosis of lung cancer. Furthermore, extracellular vesicles released into the bloodstream by many cell types are the primary sources of circulating miRNAs; this topic is covered in the following section.

**Table 6:** Brief of most related ci-miRNAs as lung tumor biomarkers and their clinical consequences [265]

miRNAs	Expression in lung tumor	Clinical correlation
miR-126	Decreased	It differs among NSCLC and normal groups and is an autonomous damaging predictive agent.
let-7	Decreased	It differs among NSCLC and normal groups and is related to shortened survivorship.
miR-145	Decreased	It is related to unfavorable prognosis in NSCLC.
miR-34	Decreased	Differentiates among LUSC and normal groups and is related to proliferation and recurrence.
miR-31	Increased	Related to LUAD patients with lymph node metastasis.
miR-205	Increased	Differentiates among NSCLC or LUSC and normal groups.
miR-221	Increased	Related to invasion NSCLC, patient recurrence, and stability to TKIs.
miR-155	Increased	It differs among NSCLC patients and normal groups and is related to OS.
miR-210	Increased	It differs among LUSC and normal groups and is related to enhanced disease-particular survival.
miR-21	Increased	It differs among NSCLC or LUAD patients and normal groups.

### Exosomal miRNA in Lung cancer

As we have already mentioned, miRNAs are delivered from the donor cells to target cells communication via exosomes (EXOs) and connect to their target mRNAs to modulate target gene expression in receiver cells. Moreover, it has been revealed that miRNAs can communicate per se with proteins [266]. The function of miRNAs discharged through EXOs is exciting, and they are being contemplated to be a robust tool for the detection and prediction of human lung cancer [267, 268]. The transcript cargo of the EXOs commonly can be different from that in the donor cells, and the EXO miRNA profile of cancer cells can also be dissimilar from that discharged via normal cells [269]. The investigation is continuing to detect the properties of EXO-released miRNAs in NSCLC. Some researchers have determined a considerable diversity among rates of miRNAs and EXOs in lung cancer patients and healthy people; however, the cancer cell miRNA templates are analogous to ci-EXO miRNAs [266, 270, 271]. In an investigation, it has been shown that miR-21- 5p and miR-29a-3p existing in lung cancer separated EXOs induce TLR-interceded NF-κβ triggering and prometastatic inflammation-inducing cancer development and advanced cancer [272]. In another investigation, the EXO miRNA profiling from plasma models has been evaluated to progress determined diagnostic equipment for LUAD. The expression template of 12 patients augmented miRNAs rates (miR-17-3p, miR-21, miR-106a, miR-146, miR-155, miR-191, miR-192, miR-203, miR-205, miR-210, miR-212, and miR-214) in cancer models was analogous to the cancer plasma-obtained EXOs and diverse template from the control samples, showing EXOs miRNAs could be related to diagnosis methods for testing this malignancy [273]. Kidd and Shumaker showed that miR-210-3p in lung cancer-obtained EXOs reduced the expression of Ephrin A3 in endothelial cells, enhancing angiogenesis [274, 275]. In another research, researchers showed that particular plasma EXO miRNAs could be used as markers displaying the frequency of NSCLC. MiR-21-5p and miR-4257 rates in plasma distinguish the recurrence of NSCLC [276]. Xiao et al. demonstrated that following miR-21 and miR-133 encapsulated in EXO deliver from the lung cancer chemotherapy-sensitive, the chemotherapy-sensitive target cells attain resistance to the medication exposure [277]. In another investigation, the EXOs miRNAs miR-378a, miR-379, miR-139-5p, and miR-200-5p were detected as robust biomarkers to identify LUAD cancer in the healthy groups. Furthermore, miR-151-5p, miR-30a-3p, miR-200b-5p, miR-629, miR-100, and miR-154-3p are robust biomarkers to diagnose LUAD from granulomas [278, 279]. Augmented rates of miR-155-5p in serum EXOs assist in the initial diagnosis of LUAD [280] (Fig. 5). Based on growing data, exosomal miRNAs from plasma play an essential role in lung cancer. Moreover, given the stable expression of EXOs in the blood and the specificity of cell expression, EXOs are expected to become accurate and sensitive biomarkers for tumor detection and prognosis [281]. Exosomal miRNAs play a critical role in cancer biology and could be potential biomarkers for cancer diagnosis. Recent research focuses on the identification of miRNAs in the secreted exosomes of the clinical samples. Owing to their excellent stability, higher specificity, and sensitivity, exosomal miRNAs could be potential biomarkers for clinical applications. However, exosomal miRNA biomarkers are still in the early discovery/development stage, and their potential value in clinical diagnostics waits to be fully explored. Understanding this type of miRNA can help in the future diagnosis and treatment of lung cancer.

## Prospects of using miRNAs in Lung cancer in preclinical and clinical studies

The first time, in 1933, Lee et al. identified miRNA (lin-4) in the nematode “Caenorhabditis elegans”, and to date, the scientist discovered different types of miRNAs. A miRNA online database such as MiRBase (http://www.mirbase.org/), which is a site for whole conceivable miRNA sequences, annotation, properties of miRNA, and target forecast information, and the 2018 version of this online database comprises 38 589 entries demonstrating hairpin forerunner miRNAs that express 48 885 mature miRNA products in 271 species [286, 287]. Up to now, an investigation was performed on www.clinicaltrials.gov and found 21 outcomes relevant to the keywords “microRNA” and “lung cancer” [288, 289]. Extremely overexpression of about 200 miRNAs out of mature human miRNAs registered in miRbase has allowed them to be included in remedial objectives. Current in vivo and in vitro experiments follow the aberrant expression of the most common miRNAs, including miR-34 and let-7 family [290]. Moreover, clinical trials assessing the diagnostic or predictive performance of miRNAs in body fluids have revealed the capability of miRNA biomarkers for non-aggressive malignancy screening. Several lung cancer-associated miRNA candidates/panels have formerly been detected [291, 292]. KRAS mutations are found in about quadrant of smoking LUAD. KRAS mutations are affiliated with opposition to currently accessible targeted therapies [293]. MiR-34a downregulates the expression of BCL2 and c-MET, which are related to the survival of cancer cells. Transduction of mimics the tumor suppressors, miR-34a and let-7, by using NLE in the mouse models showed that they are favorably targeted to lung cancer and curative advantage in NSCLC [200]. The miR-34 preclinical experiment demonstrated that this miRNA immediately inhibits checkpoint molecule programmed death ligand 1 (PD-L1) in an animal model of non-LUSC. MRX34 was used in NSCLC patients to cause the facilitated tumor infiltrating lymphocytes (TILs), cytotoxic T cells (CD8 + T-cells), and decreased CD8 + PDI + immune cells. This novel treatment technique was able to target lung cancer by controlling the p53/miR-34/PDL1 axis [279]. The positive effects of the preclinical experiment of MRX34 (miR-34a-5p mimic) to be the first curative miRNA and entered phase-I clinical studies in lung cancer patients with metastatic, such as NSCLC and SCLC that has an extension to the liver [294]. Therefore, miRNA profiling in SCLC and NSCLC patients has become the core of multiple studies. For this purpose, the generation of several methods for miRNA profile analysis is required. In addition, MRX34, a miR-34a-5p mimic liposome, was intravenously administered, but the clinical trial was ended in September 2016 owing to multiple immune adversities in patients. Moreover, no other studies of recorded clinical trials frisking the therapeutic landscapes of miRNAs in lung cancer. In addition, these advances in miRNA-based therapies are still in their early stages. This is owing to several obstacles that treatments face [59, 295].

**Fig. 6:**
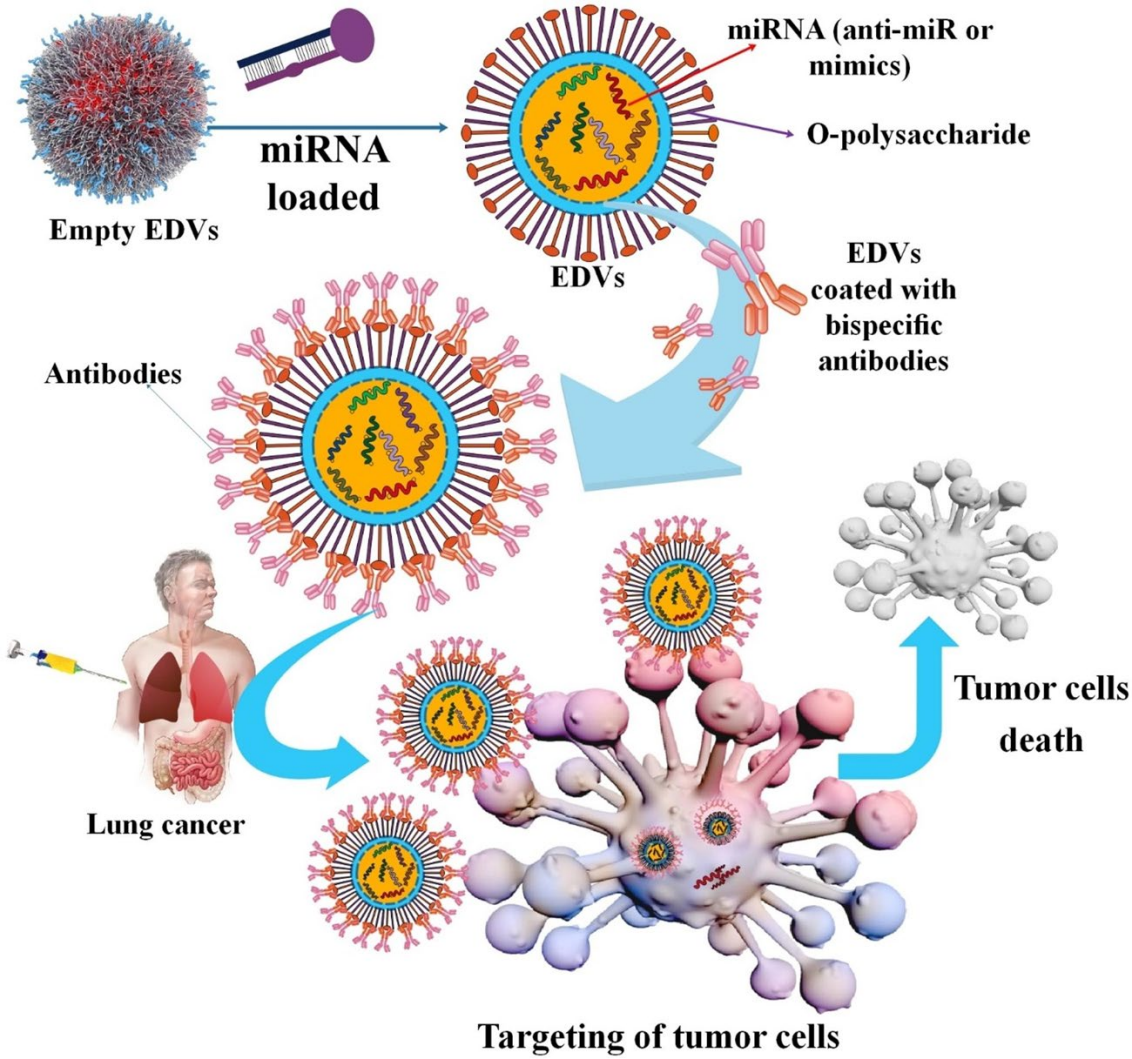
The function of EDV technology in lung cancer cells. EDV’s size is 400 ± 20 nanometers, and one EDV™ can be loaded with more than 1 million molecules of a cancer inhibitor medication. Significant concentrations of miRNAs, such as miR-16-5p mimics, are packed in empty EDVs. Then EDV™ is covered with a bispecific antibody. After this function, via the tumor microenvironment, the bispecific antibody attaches to the cancer cell membrane receptor. Afterward, the EDV™ has been absorbed, broken down inside the tumor cell, and set free miRNA. In doing this function, EDV™ Nano cells allow cancer therapies to be more powerful and tremendously less toxic

Many experiments assist in the improvement of miRNA-based therapy for clinical implementations. To utilize miRNAs as a curative agent for lung cancer, we must resolve several obstacles. These obstacles included: 1- the plurality of biological targets of miRNA cause the intense side effects in other organs, 2- miRNA mimics applied to enhancement the amounts of decreased miRNAs in disease development may be absorbed via non-target cells resulting in potential off-target efficacy. There is nodoubt that miRNA-targeting treatments are severely noteworthy to biological companies, and this therapeutic strategy is in preclinical and clinical progress for a different function in cancer patients [290, 296, 297]. Using genetically designed viral vectors to transduce miRNAs can enhance the danger depending on the integration of viral DNA into the host DNA. Charged liposomes may produce toxic and adverse events in the organs. In addition, the liposomes may have low resistance against immune response [298]. In a study, researchers developed the NP-based transfer technique as a substitute for liposomes by using EDV-Packaging. EDVs were covered with bispecific antibodies and afterward packed with miR-16-5p mimics. They are in Phase I clinical studies for NSCLS and Malignant Pleural Mesothelioma (MPM) with unsuccessful modulus treatment [299]. EDV, upon targeting the receptors available on the membrane of NSCLC, undergoes a procedure of endocytosis. They can inject better transduction miRNAs and induce the adaptive immune reaction [300, 301] (Fig. 6). MiRNAs will play a significant role in the detection and management of lung cancer in the future if more thorough and targeted research is done on them. Developing an affordable distribution mechanism that can be manufactured on a large scale is crucial. Additionally, research on many samples is necessary for this diagnosis to detect the miRNAs disrupted in lung cancer accurately. It is essential to create easily accessible, quick, accurate, and reasonably priced diagnostic techniques based on miRNAs linked to lung cancer.

## Future perspective in Lung cancer treatment and diagnosis

The NSCLC and SCLC treatment perspective has significantly progressed during the last decade. In a recent study, researchers’ analysis revealed a strong portfolio of both preclinical and clinical therapeutic entities and offered that lung cancer therapy will become even more biomarker-driven [302]. In addition, a higher understanding of lung cancer biology and the identity of oncogenic driver changes have dramatically changed the therapeutic perspective [303]. Early detection and treatment of lung cancer are vital to recovering the 5‑year survival rate for patients with lung cancer. Recently, miRNAs as encouraging biomarkers with great sensitivity and specificity for lung cancer have been investigated increasingly. In lung cancer modulatory networks, miRNAs play essential functions in the happening, development, and metastasis of SCLC and NSCLC, including oncogenic agents, tumor suppressors, and regulators. Dysfunctional miRNAs exert tumor‑suppressive or oncogenic roles in controlling cell proliferation, invasion, apoptosis, cell cycle disorder, and angiogenesis through negatively controlling target genes. Furthermore, due to its increased stability and broad practical application, miRNA may be one promising biomarker for lung cancer detection [304]. However, there are main problems in miRNA therapeutics for treating NSCLC and SCLC. The challenges lie in the tissue-particular transfer to the lung cancer cells, preserving the stability of miRNA versus nucleases and other degradative enzymes, defining efficient dosage, etc. Several miRNA delivery systems are under investigation and in preclinical assessments. It can be expected that many more new miRNA delivery systems and methods could reach and effectively complete clinical stages. Therefore, the choice of miRNA delivery systems should be devised to decrease the off-target effects of miRNA therapies in lung cancer. Several computational tools are accessible that could forecast the capability targets of miRNA, and this data could improve the science of deciding the accurate miRNA as a therapeutic agent. The future of this field lies in preparing a favorable delivery system and producing an in vivo tumor-mimicking design that could assist in clarifying different data or help in forecasting off-target effects of miRNA [305, 306]. Lung cancer is a heterogeneous disease and is caused by complex molecular aberrations. Clinically effective biomarkers should have very great sensitivity and specificity for the diagnosis of lung cancer. For example, sputum and plasma have various properties as a substitute material for molecular diagnosis of lung cancer. In particular, sputum miRNAs offer cell-based biomarkers produced from molecular alternations of the exfoliated bronchial epitheliums of large airways or main bronchial where LUSCs typically reside. In contrast, cell-free plasma miRNA biomarkers are based on diagnosing the circulating molecules per se secreted from lung tumors and floating cancer cells [307]. One of the problems in cancer diagnosis by miRNAs is that the inconsonance of miRNA panels inhibits further comparison of several investigations and decisions regarding a favorable diagnostic model. Once an advantageous model is developed, large-scale clinical trials are needed for confirmation and provision of an outstanding level of proof, which is the same urgency of miRNA investigations on biomarkers in NSCLC and SCLC [308]. The relation between miR-21 and the pathogenesis of lung cancer is a significant focus of investigation interest. miR-21 has potential clinical value in the detection and forecast of lung cancer and may be used as a successful diagnostic biomarker and therapeutic target in the future [309, 310].

## Conclusion

About 14% of the newly occurring cancers happen to be lung tumors. The principal provocations in the therapy of lung cancer are inappropriate techniques of initial diagnosis and acquired medicine resistance, which weakens the Chemotherapy advantages. Consequently, Scientists want to overcome this problem by discovering a new method with sensitive, accurate, and no side effects for treating lung cancer. Finding the function of miRNA in malignancy improvement and development has attracted our attention to miRNA as a novel therapeutic target. miRNAs expression levels differ in lung malignancy tissue compared to healthy lung tissue. In addition, ci-miRNAs, perhaps in amalgamation with other hopeful molecular markers containing epigenetic and genetic features, may be encouraging choices for quick lung cancer diagnosis. Furthermore, recent literature argues that miRNA expression varies among different subtypes of lung carcinomas. This difference helps clinicians to make use of this method for ameliorated detection. Presently, the clinical usefulness of miRNAs are lagging because of their remarkable sequence resemblance and infrequency. Moreover, miRNAs have their share of side effects. Weak infiltrates of miRNAs into the tumor tissue, unmodified miRNA antagonists, and rapid degradation and clearance of miRNA mimic blood circulation. MiRNAs, similar to ss-RNAs or ds-RNAs, contain the potential to induce immunotoxicity. Further, weak intracellular transfer and interfering free miRNA accumulation in the endosomes result in unsuccessful target genes inhibiting. These shortcomings prevent and hinder the clinical efficacy of miRNA delivery. The usage of miRNAs for targeted treatments is still in its initial periods, and only a small number of miRNAs have entered the clinical tests. By using a suitable delivery strategy, such as safe and targeted miRNA carriers (e.g., NPs, exosomes, EDVs, etc.), miRNA turns out to be an excellent therapeutic lung cancer. Further research on miRNAs can undoubtedly deepen our understanding of various malignancies, particularly lung carcinomas, and subsequently, more effective care.

## Data Availability

Not applicable.
